# MiRNA-seq-based profiles of miRNAs in mulberry phloem sap provide insight into the pathogenic mechanisms of mulberry yellow dwarf disease

**DOI:** 10.1038/s41598-018-19210-7

**Published:** 2018-01-16

**Authors:** Ying-Ping Gai, Huai-Ning Zhao, Ya-Nan Zhao, Bing-Sen Zhu, Shuo-Shuo Yuan, Shuo Li, Fang-Yue Guo, Xian-Ling Ji

**Affiliations:** 1 State Key Laboratory of Crop Biology, Shandong Agricultural University, Taian, Shandong 271018 People’s Republic of China; 2 College of Forestry, Shandong Agricultural University, Taian, Shandong 271018 People’s Republic of China

## Abstract

A wide range of miRNAs have been identified as phloem-mobile molecules that play important roles in coordinating plant development and physiology. Phytoplasmas are associated with hundreds of plant diseases, and the pathogenesis involved in the interactions between phytoplasmas and plants is still poorly understood. To analyse the molecular mechanisms of phytoplasma pathogenicity, the miRNAs profiles in mulberry phloem saps were examined in response to phytoplasma infection. A total of 86 conserved miRNAs and 19 novel miRNAs were identified, and 30 conserved miRNAs and 13 novel miRNAs were differentially expressed upon infection with phytoplasmas. The target genes of the differentially expressed miRNAs are involved in diverse signalling pathways showing the complex interactions between mulberry and phytoplasma. Interestingly, we found that mul-miR482a-5p was up-regulated in the infected phloem saps, and grafting experiments showed that it can be transported from scions to rootstock. Based on the results, the complexity and roles of the miRNAs in phloem sap and the potential molecular mechanisms of their changes were discussed. It is likely that the phytoplasma-responsive miRNAs in the phloem sap modulate multiple pathways and work cooperatively in response to phytoplasma infection, and their expression changes may be responsible for some symptoms in the infected plants.

## Introduction

Mulberry trees that have long been cultivated for sericulture are susceptible to many diseases, among which yellow dwarf disease caused by phytoplasma is one of the most devastating^1^. Phytoplasmas are wall-less, obligate intracellular plant pathogens in the class *Mollicutes*^2^ that infect several hundred economically important plants and cause devastating losses in agriculture and forestry^3^. The inability to culture phytoplasmas *in vitro* makes it difficult to characterize the plant pathogens at the molecular level, and the underlying molecular mechanisms of their pathogenicity are still poorly understood^4^.

When subjected to pathogen infection, the host plant activates sophisticated response mechanisms to reprogramme the expression of genes, proteins and metabolites^5^. The gene, protein and metabolite profiles in some host plants challenged with phytoplasmas have been investigated by differential methods^6–16^. MiRNAs functioning as negative regulators of gene expression are involved in the control of plant development and immunity^17^. Increasing evidence showed that miRNAs serve as an important mechanism for mediating gene expression during plant-pathogen interactions, and many miRNAs have been linked to resistance responses in plants^18–21^. A group of bacteria-responsive miRNAs and their target genes have been identified, and their regulatory functions have been extensively characterized in model plant species^21–29^. However, to our knowledge, only three studies have explored phytoplasma-responsive miRNAs in Mexican lime (*Citrus aurantifolia* L.), mulberry (*Morus multicaulis* Perr.) and *Paulownia fortunei*^30–32^. Although some miRNA families are conserved among various plant species, every species will have their own specific miRNAs, and the functions of these miRNAs may also be species-specific^33,34^. Moreover, different miRNAs, in addition to individual miRNAs in the same family, may be expressed differentially in various tissues and have different functions in response to the same pathogen infection^35^. Therefore, phytoplasma-responsive miRNAs have not been fully explored, and their mediating mechanisms for gene expression in response to phytoplasma are largely unknown.

Phloem is not only the major route for the translocation and distribution of organic metabolites but also an important mediator of whole-plant communication involved in whole plant events, including stress responses and long-distance signalling^36^. Recently, many miRNAs have been identified from the phloem exudates from *Brassica napus*^37^, *Malus domestica* (apple)^38^, and *Lupinus albus*^39^. Although the role of phloem miRNAs is not yet clear, some miRNAs in the phloem have been demonstrated to use long-distance signalling and have a role in mediating plant developmental patterning and stress responses^38,40–48^. In plants, phytoplasmas are restricted to the sieve elements of phloem tissues^49^. Therefore, it is reasonable to assume that the phloem is the site where the host immediate defence response against phytoplasma occurs and is involved in the coordination of the defence response at the whole plant level. Identification and characterization of phytoplasma-responsive miRNAs in the phloem sap promises to enhance our understanding of the molecular mechanisms involved in yellow dwarf disease symptom development.

In the present study, based on transcriptome information for mulberry, we employed high-throughput sequencing to profile the miRNAs in the phloem sap during the response of mulberry to phytoplasma infection. Phytoplasma-responsive miRNAs were identified, and their potential target genes were predicted. In addition, the translocation and functions of the mul-miR482a-5p involved in the response of mulberry to phytoplasma infection were discussed. The results reported here may facilitate our understanding of phytoplasma pathogenicity.

## Results

### Purity assessment of phloem sap

**Figure 1:**
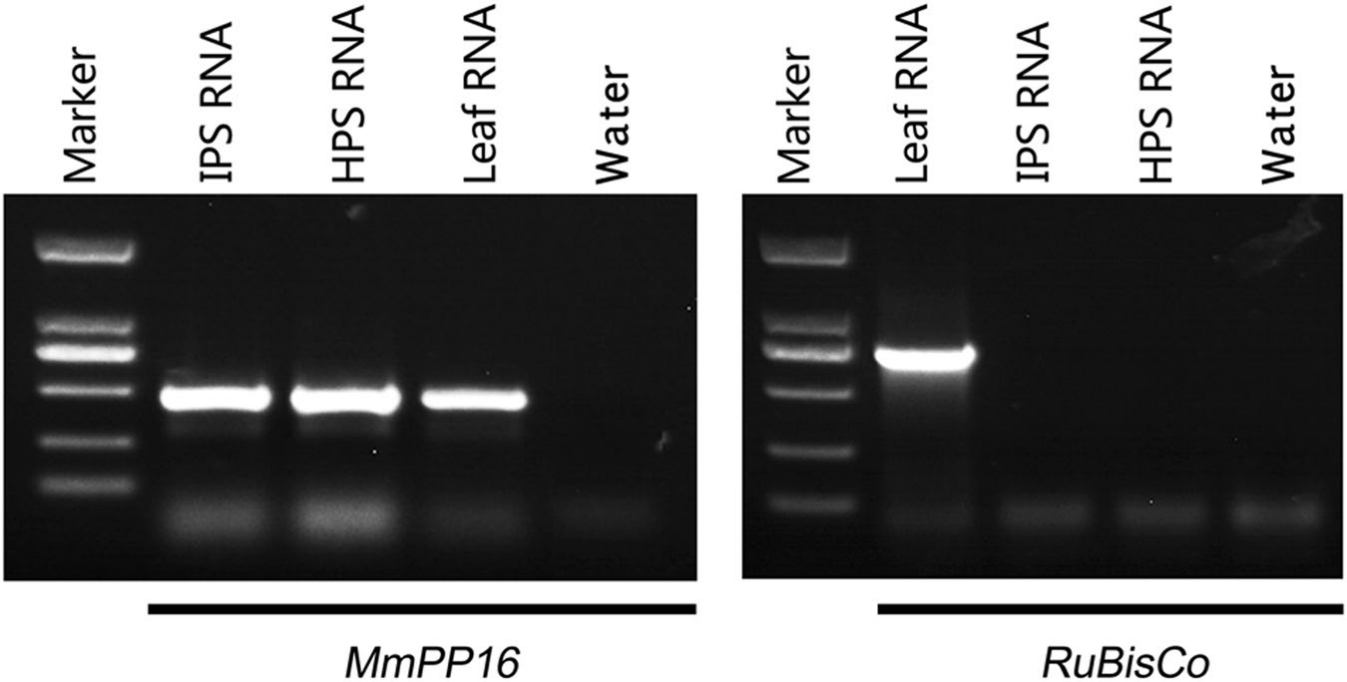
Total RNAs extracted from mulberry leaves and phloem saps were analysed by RT-PCR for the presence of *RuBisCo* and *MmPP16* mRNAs. IPS, phloem sap sampled from infected trees. HPS, phloem sap sampled from healthy trees. The gels used were cropped from different gels showed in the Supplementary Figure 1.

To identify phloem-enriched sRNAs and ensure that the sRNAs identified in phloem sap did not result from contamination during sampling, the frequency of the ribulose bisphosphate carboxylase oxygenase (RuBisCo) large subunit gene that would be expected to in leaves, but not the sieve element–companion cell complex, was determined to assess the purity of the phloem sap sampled. The results detected no *RuBisCo* mRNA in the phloem sap samples, but this mRNA was clearly present in leaf tissue. Meanwhile, phloem-specific *MmPP16* mRNA was detected in phloem sap samples (Fig. 1). This indicates that contamination from surrounding tissues in the collected phloem sap samples was very low.

### Overview of small RNA in phloem saps

**Figure 2:**
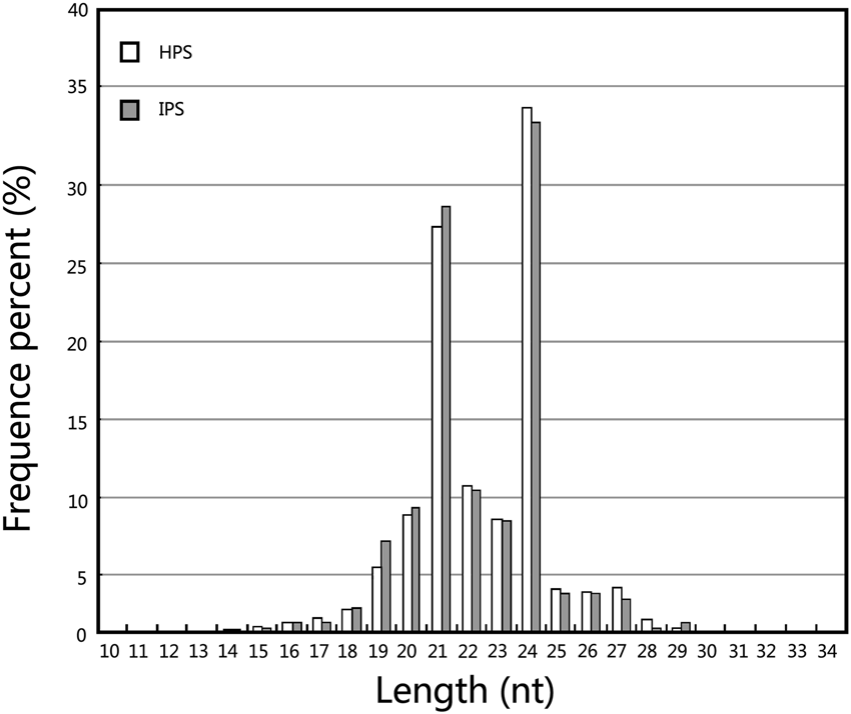
Length distribution of small RNA in mulberry phloem sap sRNA libraries. IPS, phloem sap sampled from infected trees. HPS, phloem sap sampled from healthy trees.

To examine the phytoplasma-responsive miRNAs in phloem sap, the sRNA libraries were constructed from phytoplasma-infected and healthy mulberry phloem saps and subjected to Solexa deep sequencing. After removing the rRNA, tRNA, and degradation products from the matching sequences, the remaining clean small RNA sequences were aligned with the miRNA precursors/mature miRNAs in the miRBase database v21.0, and 86 known miRNAs members belonging to 78 families were identified (Table 1). All non-annotated sRNA sequences were mapped to our mulberry transcriptome database, and 19 sequences were found to perfectly match the transcriptome sequences. The mapped RNAs were able to fold into hairpin structures and had negative folding free energies (from −21.0 to −95.65 kcal mol^−1^ with an average of about −49.05 kcal mol^−1^) according to Mfold; these values were lower than the folding free energies of rRNA (−33 kcal mol^−1^) and tRNA (−27.5 kcal mol^−1^)^50^. Therefore, these mapped RNAs were identified as candidate novel miRNAs (Table 2). Interestingly, some miRNA-3p sequences, such as mul-miR160b-3p, mul-miR166h-3p and mul-miR169p-3p, were identified, but the corresponding miRNA-5p sequences were not detected. This may indicate that these miRNA-3ps may be the authentic miRNAs. The size distribution of the small RNA sequences identified in the two libraries showed that more than 80% of the mapped small RNAs were 20–24 nt long, with 24 nt and 21 nt as the major size groups (Fig. 2).

**Table 1:** Profiles of conserved miRNAs in mulberry phloem saps.

MiRNA-name	Sequence (5′-3′)	Normalized value		Fold-change (log_2_ IPS/HPS)	P-value	Significance lable
		IPS	HPS			
mul-miR1134	CAGAACGAAGAAGAAGAAGAAGA	22.10	22.34	−0.02	0.90042163	
mul-miR1223e	UUGAGAUGUCAUGCACCACUCUG	1.58	5.11	−1.69	1.18E-06	**
mul-miR1310	GAGGCAUCGGGGGCGCAACG	44.79	24.47	0.87	5.19E-18	
mul-miR1511	ACUAUGCUCUGAUACCAUGUUAA	1.25	5.35	−2.10	9.19E-09	**
mul-miR1520m	AAUUCAAACUGAGAUGUGACAUU	132.28	124.54	0.09	0.08960825	
mul-miR156a-5p	UGACAGAAGAGAGUGAGCAC	20.68	7.55	1.45	8.14E-19	**
mul-miR157a-5p	UUGACAGAAGAUAGAGAGCAC	881.9	44.69	4.30	0	**
mul-miR159a-3p	UUUGGAUUGAAGGGAGCUCU	84.07	158.84	−0.92	1.63E-64	
mul-miR160a-5p	UGCCUGGCUCCCUGUAUGCCA	1.97	0.75	1.39	0.00994309	**
mul-miR160b-3p	GCGUAUGAGGAGCCAUGCAUA	22.44	15.66	0.52	0.00010904	
mul-miR164a-5p	UGGAGAAGCAGGGCACGUGCA	3.54	8.42	−1.25	5.02E-07	**
mul-miR165b-5p	GAAGUGUUCGGAUCGAGGC	9181.72	2752.65	1.74	0	**
mul-miR166a-3p	UCGGACCAGGCUUCAUUCCCC	2068.07	1700.73	0.28	2.47E-98	
mul-miR166h-3p	UCGGACCAGGCUUCAUUCCC	1899.18	1541.57	0.30	4.99E-102	
mul-miR167d-5p	UGAAGCUGCCAGCAUGAUCUG	2.25	1.81	0.32	0.44192478	
mul-miR168a-5p	UCGCUUGGUGCAGGUCGGGAA	161.64	163.80	−0.02	0.67470362	
mul-miR168a-3p	CCCGCCUUGCAUCAACUGAAU	1.75	2.44	−0.48	0.24497579	
mul-miR169b	CAGCCAAGGAUGACUUGCCGG	1.75	2.67	−0.61	0.12748684	
mul-miR169p-3p	GGCAUAUGAUCAUCUUGGGGCUAG	3.84	10.86	−1.50	6.76E-11	**
mul-miR171g-5p	UUUUGGAUGGCUCAACACG	20.10	39.26	−0.97	1.48E-18	
mul-miR172a-3p	AGAAUCUUGAUGAUGCUGCAU	21.85	38.16	−0.80	1.15E-13	
mul-miR172e-3p	GAAUCUUGAUGAUGCUGCAU	21.85	38.94	−0.83	1.04E-14	
mul-miR1856	UACGUAGAGGCGGAUUCGUA	88.82	43.58	1.03	2.35E-44	**
mul-miR1858a	GAGCGGAGGACUGUAGUGGGUGC	37.11	93.62	−1.33	2.27E-69	**
mul-miR2108b	GUUAGAUGUGAUUGUUUGUGAG	329.36	282.59	0.22	2.99E-11	
mul-miR2118-5p	GUCGAUGGAACAAUGUAGGCAAGG	562.31	157.74	1.83	0	**
mul-miR2199	UGAUAACUCGACGGAUCGC	61232.99	54719.33	0.16	0	
mul-miR2670f	GGGUCUGUUUGGUUGGGGGA	89.83	49.64	0.86	1.89E-33	
mul-miR2867-3p	CCAGGACGGUGGUCAUGGA	89.58	77.81	0.20	0.00138453	
mul-miR2873b	AUUGGCUGGAGAUAUUGGUAUG	30.36	26.98	0.17	0.11724709	
mul-miR2911	GCCGGGGGACGGACTGGGAA	153874.88	140579.96	0.13	0	
mul-miR2916	GUUGGGGGCUCGAAGACGAUCAGA	6604.64	5708.74	0.21	3.21E-177	
mul-miR319a	UUGGACUGAAGGGAGCUCCC	1.33	7.63	−2.52	1.56E-14	**
mul-miR3630-3p	GGGAAUCUCUCUGAUGCA	0.50	1.65	−1.72	0.00597169	**
mul-miR3630-5p	GCAAGUGAUGAUAAACAGACA	2.09	3.46	−0.73	0.04145473	
mul-miR390a-5p	AAGCUCAGGAGGGAUAGCGCC	25.19	10.31	1.29	4.65E-19	**
mul-miR390a-3p	CGCUAUCUAUCCUGAGUUUCA	3.17	1.97	0.69	0.06255943	
mul-miR393a-5p	UCCAAAGGGAUCGCAUUGA	1.50	2.12	−0.50	0.25832055	
mul-miR396a	UUCCACAGCUUUCUUGAACUG	0.92	1.73	−0.92	0.08385227	
mul-miR396b-3p	GCUCAAGAAAGCUGUGGGAGA	4.50	6.53	−0.54	0.03282992	
mul-miR397a-5p	UCAUUGAGUGCAGCGUUGAUG	1.33	0.16	3.08	0.00046347	**
mul-miR398a-5p	GGCGUGACCCCUGAGAACACAAG	1.08	2.75	−1.34	0.00276708	**
mul-miR408b-5p	CAGGGAACGGACAGAGCAUGG	61.30	63.57	−0.05	0.47719536	
mul-miR4403	ACGGCACAAACACGACACGAGCAC	3.75	2.52	0.58	0.08327133	
mul-miR4414a-3p	AUCCAACGAUGCAGGAGCUAGCC	3.67	7.32	−0.99	0.00010668	
mul-miR4414a-5p	AGCUGCUGACUCGUUGGUUCA	47.54	75.37	−0.66	9.44E-19	
mul-miR447b-5p	ACUCUCACUCAAGGGCUUCA	1.67	0.79	1.08	0.04888033	*
mul-miR472b-3p	UUUUCCCAACACCACCCAUACC	22.77	19.51	0.22	0.07812493	
mul-miR473a-5p	ACUCUCCCCCUUAAGGCUUCCA	70.81	115.18	−0.70	1.95E-30	
mul-miR477c	CUCUCCCCCUUAAGGCUUCC	77.90	139.01	−0.84	1.32E-48	
mul-miR482a-3p	UUCCCAAGGCCGCCCAUUCCGA	217.77	160.57	0.44	3.91E-25	
mul-miR482a-5p	GGAAUGGGCUGUUUGGGAAGA	2140.30	964.91	1.15	0	**
mul-miR5021	GAGGGAGAAGAAGAAGAAGA	47.04	54.12	−0.20	0.01340026	
mul-miR5039	CCCUAUUUUUAAUCGUUGGA	0.92	1.42	−0.63	0.26283984	
mul-miR5054	GUGCCCCACGGUGGGCGCCA	16.99	1.25	3.76	1.51E-44	**
mul-miR5059	CGGGCCUGGCGCACCCCA	940.30	1455.27	−0.63	9.97E-301	
mul-miR5072	GUUCCCCAGUGGAGUCGCCA	72.69	2.92	4.64	3.41E-214	**
mul-miR5077	UUCACGUCGGGUUCACCA	12.51	28.32	−1.18	1.67E-18	**
mul-miR5085	AAGGACAUUUGGUUGUGGCUC	129.36	187.63	−0.54	1.08E-30	
mul-miR5139	GUAACCUGGCUCUGAUACCA	2.25	1.10	1.03	0.02729391	*
mul-miR5224a	UUGAUGGACAUGAAGACGUUAU	4.84	4.09	0.24	0.38021350	
mul-miR5266	CGGGGGACGGACUGGGGC	25.35	36.27	−0.52	1.02E-06	
mul-miR5279	GGAACCUCGGAUGAUCGGUUA	6.17	9.91	−0.68	0.00105543	
mul-miR5293	GGAGGAAGUGAGAAGAAGAAGA	9.34	10.78	−0.21	0.2625989	
mul-miR529-3p	GCUGUACCCCCUCUCUUCUC	1.83	0.55	1.74	0.00316862	**
mul-miR529b	AGAAGAGAGAGAGUACAGCUU	21.35	5.35	2.00	1.85E-29	**
mul-miR5368	GGGACAGUCUCAGGUAGACAGUU	1.17	0.63	0.89	0.16322128	
mul-miR5386	CGUCAGCUGUCGGCGGACUG	33.70	47.28	−0.49	1.11E-07	
mul-miR5568f-3p	GUCUGGUAAUUGGAAUGAG	447.29	559.12	−0.32	3.04E-35	
mul-miR5641	UGGAACGAACAGAGAUAGAAUUA	3.17	2.60	0.29	0.40238294	
mul-miR5813	ACAGCAGGACGGUGGUCAUGGA	29487.21	24982.87	0.24	0	
mul-miR6030	UCCCCCAACCAAACAGACCCU	77.98	114.39	−0.55	2.53E-20	
mul-miR6150	AGUUUGUUUGAUGGUACUUGC	764.73	1088.74	−0.51	1.54E-154	
mul-miR6180	AGGGUCGGAGGAAAGAGGGCC	2.00	0.63	1.67	0.00267402	**
mul-miR6191	AUAAUUUGUCUGGUUAUGAA	19.77	22.34	−0.18	0.16412556	
mul-miR6196	GAGGACAGGAGUAGAGAGGA	5.59	3.07	0.86	0.00251337	
mul-miR6214	CACGACACGAGCUGACGACA	5.09	0.01	8.99	6.91E-20	**
mul-miR6235-5p	UGUGAGAGAAAAUACUGUAGCGA	115.68	110.14	0.07	0.19506665	
mul-miR6300	GUCGUUGUAGUAUAGUGGU	5283.44	18100.50	−1.78	0	**
mul-miR6478	CCGACCUUAGCUCAGUUGG	41.53	9.20	2.17	4.78E-62	**
mul-miR845a	CGGCUCUGAUACCAACUGUGACG	6.92	6.37	0.12	0.59522396	
mul-miR854a	GGAUGGGAUGGAGGAGGAG	30.19	33.36	−0.14	0.16388474	
mul-miR894	GUUUCACGUCGGGUUCAC	46.96	97.32	−1.051	6.61E-50	**
mul-miR952b	AACGAGGAUCCAUUGGAG	911.10	888.21	0.04	0.05791621	
mul-miRn10-3p	AGGUGCAGAUGCAGAUGCAGG	6.6723	0.01	9.38	7.50E-26	**
mul-miRn12-3p	UCUUGCCGAGACCUCCCAUA	33.6116	28.2432	0.25	0.016399105	

IPS, phloem sap sampled from infected trees. HPS, phloem sap sampled from healthy trees.

**Table 2:** Novel miRNAs in mulberry phloem saps by Illumina sequencing.

MiRNA-name	Sequence (5′-3′)	Precursor sequence (5′-3′)	Energy (kcal mol^-1^)
mul-miRn21-3p	GAGCAGUGCGGAGUAGCUGAG	GGUUCCGGCUGUUGCGUUGGAACUGAAUGUCUUAUUUAUUCACUUCAUCAUUUAUUUAUUUAACGCUUUCUUAUUUAUUUAUAAAGUUAUUUCAAAUUAUAUCCUACUGGACCUUUUACUCACGUUUUAUUGUUUUAAAAAUGUUAACCCCUCUCUUGAGACUCUAAGAGCAGUGCGGAGUAGCUGAGUUG	−33.60
mul-miRn22-5p	CAGCGAACUAAACGGGCCCU	AUCAGCGAACUAAACGGGCCCUUAAACUUUCGUUUUUUCACCUCAUCAUUCUGUACUUGUUAACUUCUGUAGAAAUCUUAAAAAAUUCAAUUAAACACUCAUUUUGUCCCCACUCAGAGCUGAUAAUUUAAAAGUUUAAAAAUUCUAGCCAUACUCACUUAGGGCGUGUUUGGUUCGGGGGA	−35.84
mul-miRn23-5p	UGAGGAUGUAUCAGAAGAUAG	AAAGGGUCCCUGAGGAUGUAUCAGAAGAUAGUGCAGAUAUUUGGUUUUGAUAGGCAUUCUUGUUCUGGAGUAUGCUUUUGCUUAUCCUUCCCCCACUACAUUCGCAUUGCUGGAUCUGCUUGAGGAAACUAAAACCAACAAUAAUUUUAGUUAAAGUAUUCUGCUGUUGUGGAAUAUUGGUUGACACAUGAGAUAUCUCCCUUUCUUUCACCGUCAAAGGCAGAACUCUCUUGUUCCCACUUUCCUCUAACACUUCAGCUCCAGCUGUAGAAGAGAAAGUAUCUCGCUUAGCCACACCAAUGUAUUUUGACUUGCCUCUGACAGAGGUGUUUUUGAUAGCAUCUCUCAAAACAUACAUUUUAGUGGUAUUCC	−95.65
mul-miRn24-5p	AAGCUAGCUGUGUGGAUGAUA	UAUGCAACAAAAGCUAGCUGUGUGGAUGAUAUUAAUUGUCGUUUUAGCGGCAGCGUGUUUCGUUGUCGC	−21.80
mul-miRn25-3p	UUCCAAAUCCACCCAUGCCCAC	UUUGAGCUUUAUGAAGUUGUCGGGCCUGGGAGGUUUGGUAGGAGUAAUAAGUAAUUACCAUUUAGUUUUUUGUUCACUUAAUUGAUAUUAUAAUUGUAUGUUUUAAUUUAGUUCUCCUUCCAAAUCCACCCAUGCCCACAAUUUCCUCAGGCUUCUCUC	−51.80
mul-miRn26-5p	GCUUCCUCGGAGACGGCGCACG	AGAAUAACAACAAAUCGGCAGCUUCCUCGGAGACGGCGCACGACAGCAAGAAGGGUGGGUCGUCCUCGGGAAGCGGAGAGCAGGCGGCGGCGCCG	−37.00
mul-miRn27-5p	CAGACAUUGAGUGGGGGAGG	UGAUAUUUUCAGCCUGUAAUCAGACAUUGAGUGGGGGAGGAAGAGAAGAUCUCGUUACCGGCGGGUCGACCCGGAUAAACCGCUGGAAAAUGACGGUUUUGUUCCUUGCCACGCGGCGGCCGGCGAUCGGUGCCAGCCAUGGUAGCCGCGGUCUCAUCUCUCUCCCGCGCUUUGUCUGAGAAACGGCCAGUCUGAGCCC	−82.7
mul-miRn28-3p	GGACUUUAUGGACCCGUCGGUG	CGGGCAAUGCUGUGAACGGUCAGAUCCCGCCGGCCGACCGGUCUGUGAGUCCUUUGUUACCGGACUUUAUGGACCCGUCGGUGUGCUAUGUCC	−37.9
mul-miRn29-5p	GUGGAUCAAGAACUGGAGGC	UUUGCAACAUGUGGAUCAAGAACUGGAGGCAAAGGUUACUGCUUCAUUUGCACCACAGAGAUCUCAAGUGGCACAACCUCCUGCAACCAAAGGUUCUUAUUCACAGGUUGGAUU	−35.1
mul-miRn30-3p	UCCAGAAGCAAUCGUACGGGA	CGUGGAGCACCCUGUUCCUGUAGUUGCUCCUGGAUCUGCUAAGAAUCUCUCUGAAGUCAAAAUUAAUCCAGAAGCAAUCGUACGGGAAGGACACACU	−27.9
mul-miRn31-3p	AUGCACUGCCUCUUCCCUGGC	AGAGGGGGUCAAAAAGCAGAAUAAGGCAGGGAACGGACAGAGCAUGGAUGGAGCCUUCAACAGAAGAAGGAAUGCUGUUGUGGCUCUACUCAUGCACUGCCUCUUCCCUGGCUGUGCCUCUC	−56.7
mul-miRn32-5p	GGAAUGUUGUCUGGCUCGAGG	AAUCCCGCUAAGAAGUCUUUGUUUAAGAGCUAUAGACUAUAAAGUAAGGGAAUGGGACCCCGACGGGAAUGUAUCCCAAGCAGCGGAGUAAGUUCACUCUUUGGUAAGCUUAGGCGCCUAAAUGUCUGAGUUCGGAAAGCAGAGUAAGCGGCGAUC	−45.0
mul-miRn33-3p	GGGAGAAAGAGGAAAAUAGGC	UUUGUUUUUGUUUUUUUUUUUAAUAUCACAAGAUUCACAAGUCACAACCCUGCUUGUUAGAGCACAGACACAAGCAACUGUAGGUGGACUAAGUAGCCAUGGGCCAGGCUGAGAGCGGUAUCAUGAGGCCUCGCAGAGUAGCUUCAAUGCAUUGGAUGCAUUUCCAACGUAAAAAAUUUCACUUUUAAGCGUGUAAUUUUUAAAUAAUUUUUAAGAAUUUUUUUGGGAGAAAGAGGAAAAUAGGC	−55.6
mul-miRn34-5p	GGGAGCUGAGUUGAUGAGCA	GGACUGCUAAACAAGCUCGUCUCUCGCUCCCUCUCCGUCGCUGGAAAAUGGCAGCAGCAACAGCUCCGCCGUCUUAACAUCCAUGAAUAUCAGGGAGCUGAGUUGAUGAGCAAAUGUGGGAU	−43.6
mul-miRn35-5p	GCAGAAGAGUCAGAGCUUUGA	AGGUAGUUUUGCAGAAGAGUCAGAGCUUUGAUUUGAAUCUCAGAAAAAAUAAAAACCGAGAAAGAAAAAAGAAAUGGCGAGCCCGAAAUCCGGCAGCCAAAACGACGGCGUUCCGUGCGACUUCUGCAGCGAGCAAACGGCGGUGCUGUACUGCAGAGCCGACUCGGCGAAGCUCUGCCUCUUCUGCGACCAGCACGUCCA	−75.2
mul-miRn36-3p	GCUGAAGCUGGGGUGGGGCC	GCGGCGGCUCUGGUGCUCCACACCUUCUUCAGCUGGCUGCUGAUGCUGAAGCUGGGGUGGGGCCUUGCCGGCGG	−41
mul-miRn37-3p	GGACGGCAUCGAUCGGAGCUC	CGGCGAGGGAGCUCCGACCGAAGCUUCCUCUUGGCGAUGGACGGCAUCGAUCGGAGCUCUUGCUUCGCU	−37.2
mul-miRn38-3p	GACUGAAAGCGGACCUGGUGGUG	GAAGAUUUGUAUUUGGCCGCCAGGUCCACCUUCAGUCUUCUUCAAAGACCUUCGUUGCUGCCACACAGCCAGCUUUGGUUUCAAGGACUGAAAGCGGACCUGGUGGUGAUAAUUCAAA	−49.5
mul-miRn39-5p	UCGACCAGCCGAGUAGAAGUA	AUAUUGGAUCUCGACCAGCCGAGUAGAAGUAAGUAUCUCAUUUCCCUCGAUUGUUACUUCUACUCGGCUGGUAGAGAUUCAAUGUU	−50.6

### Expression profiling of miRNAs in response to phytoplasma-infection

**Figure 3:**
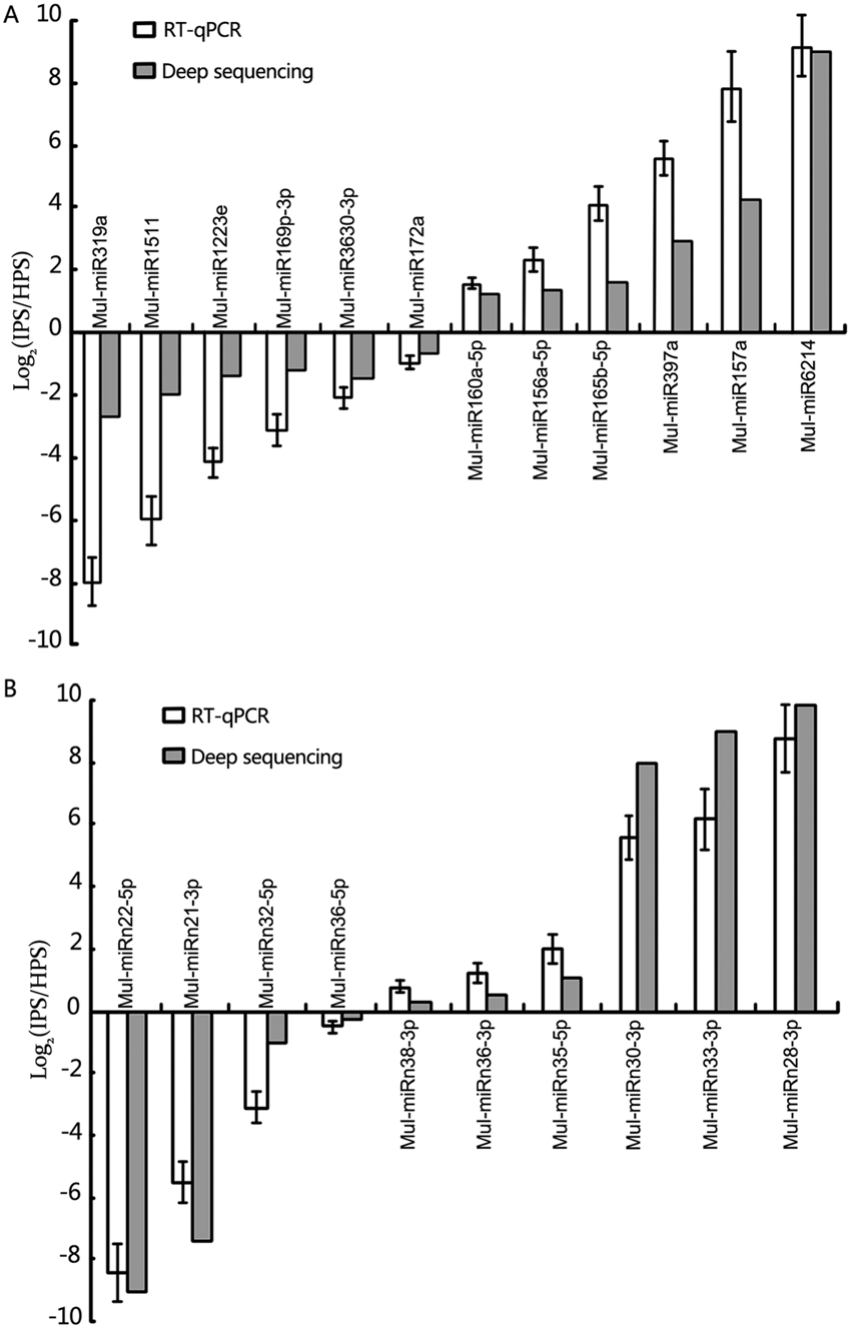
Verification of selected miRNAs from deep sequencing by RT-qPCR. (**A**) Conserved miRNA abundance analysis by RT-qPCR. (**B**) Novel miRNA abundance analysis by RT-qPCR. Relative miRNA abundance was evaluated using comparative Ct method with U6 as the reference. Log_2_ values of the ratio of phytoplasma-infected samples to healthy samples are plotted. Values are given as the mean ± SD of three experiments per group. IPS, phloem sap from infected trees. HPS, phloem sap from healthy trees.

The profiles of some miRNAs differed between the healthy and infected phloem sap libraries. A total of 30 known miRNAs and 13 novel miRNAs were found to be differentially expressed between phytoplasma-infected and healthy mulberry phloem sap libraries (Tables 1, 3). These differentially expressed miRNAs were considered phytoplasma-responsive miRNAs, among which 15 miRNAs decreased and 28 miRNAs increased significantly in the infected phloem saps (P < 0.05, fold 2.0). These phytoplasma-response miRNAs consist of not only highly expressed miRNAs such as mul-miR166a, mul-miR166h-3p, mul-miR2199, and mul-miR5813 but also low-abundance miRNAs such as mul-miR160a, mul-miR167d-5p, mul-miR3630-3p, mul-miR3630-5p, and mul-miR396a. Although both the miRNA-5p and miRNA-3p of some miRNAs were detected, only miRNA-5p or miRNA-3p changed during phytoplasma-infection. This strongly suggested that single-strand miRNAs, and not miRNA-5p/miRNA-3p duplexes, are the phytoplasma infection-relevant molecular species. To validate the miRNA expression differences revealed by the sequencing experiments, we performed RT-qPCR analysis for 12 known miRNAs and 10 novel miRNAs covering different expression patterns. The results obtained by RT-qPCR showed a very strong correlation with read frequencies, demonstrating that our sequencing data are quantitative and reliable (Fig. 3).

**Table 3:** Expression profiling of novel miRNAs in mulberry phloem saps.

No.	MiRNA-name	Normalized value		Fold-change (log_2_ IPS/HPS)	P-value	Significance lable
		IPS	HPS			
1	mul-miRn21-3p	0.01	1.8095	−7.50	2.38E-07	**
2	mul-miRn22-5p	0.01	4.7203	−8.88	5.02E-18	**
3	mul-miRn23-5p	1.6681	0.01	7.38	5.12E-07	**
4	mul-miRn24-5p	2.3353	0.01	7.87	1.58E-09	**
5	mul-miRn25-3p	5.7548	5.507	0.063	0.793593809	
6	mul-miRn26-5p	1.7515	0.9441	0.89	0.085551202	
7	mul-miRn27-5p	20.0168	12.5088	0.68	3.39E-06	
8	mul-miRn28-3p	9.7582	0.01	9.93	1.82E-37	**
9	mul-miRn29-5p	2.0017	0.01	7.65	2.84E-08	**
10	mul-miRn30-3p	2.4187	0.01	7.92	7.66E-10	**
11	mul-miRn31-3p	0.9174	1.8881	−1.04	0.044254454	*
12	mul-miRn32-5p	15.2628	34.9303	−1.19	6.62E-23	**
13	mul-miRn33-3p	4.8374	0.01	8.92	6.04E-19	**
14	mul-miRn34-5p	1.0842	1.6521	−0.61	0.237320172	
15	mul-miRn35-5p	1.7515	0.7867	1.15	0.03366659	*
16	mul-miRn36-3p	1.7515	1.3374	0.39	0.410468979	
17	mul-miRn37-3p	1.8349	0.01	7.52	1.21E-07	**
18	mul-miRn38-3p	1.7515	1.4948	−0.22863852	0.617178011	
19	mul-miRn39-5p	65.5551	38.6279	−0.76306477	1.07E-20	

IPS, phloem sap sampled from infected trees. HPS, phloem sap sampled from healthy trees.

### Phytoplasma-responsive miRNAs related to diverse biologic processes

**Figure 4:**
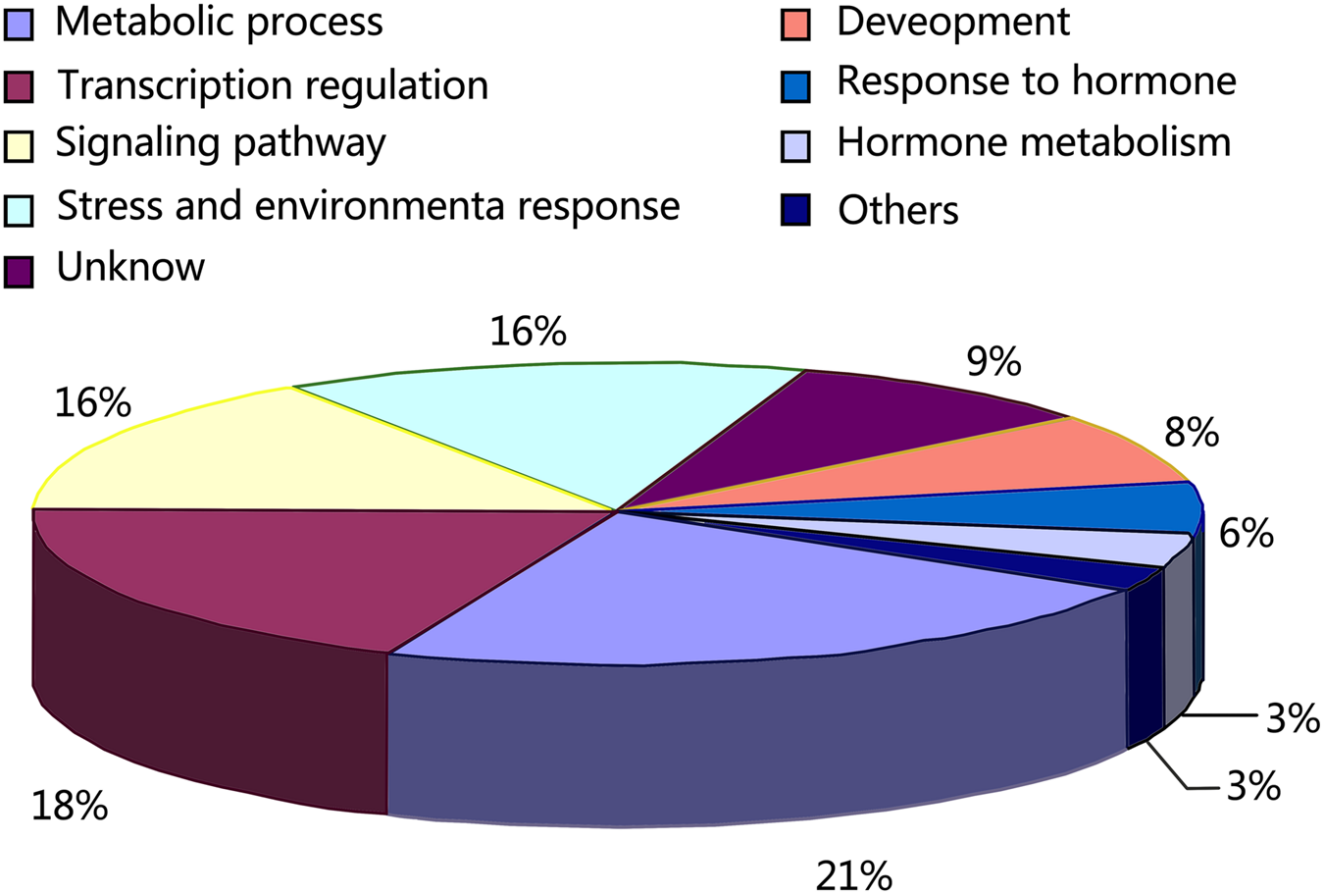
Percentage distributions of predicted target genes for differentially expressed phloem sap miRNAs in various categories.

To understand the biological functions of phytoplasma-responsive miRNAs reported here, the target genes of these miRNAs were predicted and subjected to Gene Ontology (GO) analysis. Together, 131 target genes of 36 phytoplasma-responsive miRNAs were predicted and classified into nine categories according to their ontologies in Arabidopsis, based on KEGG functional annotations (Tables 4, 5; Fig. 4). The categories included metabolic process, transcription regulation, signalling pathway, stress and environmental response, development, response to hormones and hormone metabolism. Interestingly, 16% of target genes were found to be associated with signalling pathways. This indicated that many miRNAs in the phloem sap were associated with signal transduction and that diverse signalling pathways were involved in phytoplasma infection in the infected plants. In addition, many genes homologous to the sequences of unknown functions were predicted as targets of phytoplasma-responsive miRNAs. Further analyses of these genes and miRNAs may reveal new biological functions for phloem. Thus, these phytoplasma-responsive miRNAs in the phloem saps might be related to diverse biologic processes, and the regulatory networks involved in the response to phytoplasma-infection in mulberry were intricate.

**Table 4:** Predicted targets of the differential conserved miRNAs.

MiRNA-name	Putative GO_process	Predicted target annotations in mulberry transcriptome data
mul-miR1223e	Hormone metabolism	O-fucosyltransferase
	Metabolic process	Glutaredoxin
	Metabolic process	S-adenosyl-L-methionine-dependent methyltransferases superfamily protein
mul-miR1511	Unknow	Transposable
	Stress response	Trichome
mul-miR156a	Transcription regulation	Squamosa promoter binding protein-like 7
	Transcription regulation	Squamosa promoter binding protein-like 9
	Transcription regulation	Squamosa promoter binding protein-like 10
	Signaling pathway	Protein kinase superfamily protein
	Signaling pathway	Cysteine/Histidine-rich C1 domain family protein
	Metabolic process	Putative pyridine nucleotide-disulphide oxidoreductase
	Metabolic process	UDP-glycosyltransferase-like protein
mul-miR157a	Transcription regulation	Squamosa promoter binding protein-like 7
	Transcription regulation	Squamosa promoter binding protein-like 10
	Transcription regulation	LIM domain-containing protein
	Unknown	Unknown protein
	Hormone metabolism	Galactose oxidase/kelch repeat superfamily protein
	Signaling pathway; Development	F-box family protein
	Stress response	Plant invertase/pectin methylesterase inhibitor superfamily protein
	Metabolic process	Dioxygenase-like protein
mul-miR160a	Auxin signaling; Transcription regulation	Auxin response factor 10
	Auxin signaling; Transcription regulation	Auxin response factor 16
	Auxin signaling; Transcription regulation	Auxin response factor 18
	Transcription regulation	NAC domain containing protein 1
	Transcription regulation	NAC domain containing protein 6
	Unknown	Unknown protein
	Signaling pathway	CBL-interacting protein kinase
mul-miR165b-5p	Metabolic process	Methyl esterase 17
	Stress response	Leucine-rich repeat receptor-like protein kinase
	Development	Embryo defective 1379 protein
mul-miR1856	Metabolic process	Glucan synthase-like 3
mul-miR1858a	Metabolic process	Transmembrane amino acid transporter family protein
	Stress response	Major facilitator superfamily protein
mul-miR2118-5p	Secondary metabolitic process; Environmental responses	Cytochrome P450 like_TBP
mul-miR319a	Transcription regulation	R2R3-MYB transcription factor
	Transcription regulation	Transcription factor MYB811
	Defense response	Disease resistance protein
mul-miR3630-3p	Signal transduction	Leucine-rich receptor-like protein kinase
	Signal transduction	Leucine-rich repeat transmembrane protein kinase-like protein
	Transcription regulation	Smg-4/UPF3-like protein
	Secondary metabolitic process	Lycopene beta-cyclase
	RNA process	Endoribonuclease
	Unknown	Uncharacterized protein
mul-miR390a	Auxin signaling; Development	TAS3/TASIR-ARF (TRANS-ACTING SIRNA3)
	Signal transduction	Protein kinase superfamily protein
	Signal transduction	Leucine-rich repeat protein kinase-like protein
mul-miR397a	Metabolic process; Stress response	Laccase 2
	Metabolic process	Laccase 11
	Transcription regulation	CLP protease proteolytic subunit 3
	Response to stress	TRICHOME BIREFRINGENCE-LIKE 14
	Signal transduction	Protein kinase superfamily protein
mul-miR398a-5p	Metabolic process; Transcription regulation	Purple acid phosphatase 14
mul-miR447	Metabolic process	P-loop containing nucleoside triphosphate hydrolases superfamily protein
	Microtubule-based process	ATP binding microtubule motor family protein
mul-miR482a-5p	Transcription regulation	Regulator of chromosome condensation family protein
	Metabolic process	Trehalose 6-phosphate synthase
	Metabolic process	Inositol 1,3,4-trisphosphate 5/6-kinase family protein
mul-miR5072	Metabolic process	Heteroglycan glucosidase 1
mul-miR5077	Signal transduction	Calponin domain-containing protein
	Metabolic process	Slpha-rhamnosidase-like protein
	Metabolic process	ATP-sulfurylase precursor
	Defense response	Guanylate-binding-like protein
	Unknown	Uncharacterized protein
mul-miR5139	Unknown	Transposable element gene
	Transcription regulation	RNA-dependent RNA polymerase family protein
mul-miR529b	Transcription regulation	SPL domain class transcription factor
	Transcription regulation	Squamosa promoter binding protein-like 9
	Development and environmental responses	Pentatricopeptide repeat (PPR) superfamily protein
	Responses to biotic stress	Glycine/proline-rich protein
	Response to jasmonic acid and wounding	Inosine-uridine preferring nucleoside hydrolase family protein
	Responses to viral infection	Cysteine-rich repeat secretory protein 60
	Signal transduction	Leucine-rich repeat protein kinase family protein
	Metabolic process; Environmental responses	3-ketoacyl-CoA synthase 19
	Development, response to auxin	SAUR-like auxin-responsive protein family
	Unknown	Hypothetical protein
mul-miR529-3p	Response to stress	ARM repeat-containing protein-like protein
	Response to stress	Sensitive to freezing 6
	Development	Growth-regulating factor 2
	Metabolic process	UDP-glucosyl transferase 75B2
	Metabolic process	RING/U-box superfamily protei
	Signaling pathway	Shikimate kinase 1
mul-miR6180	Metabolic process	Wax synthase isoform 1
	Response to stress	Class III peroxidase
	Metabolic process	Acyl-CoA sterol acyl transferase 1
mul-miR6300	Response to biotic and abiotic stresses,	Glycosyl hydrolase family 1 protein
	Hormone-mediated signaling pathway	Leucine-rich repeat receptor-like protein kinase
	Unknown	Predicted protein
mul-miR894	Response to auxin and ethylene; Transcription regulation	Auxin and ethylene responsive GH3-like protein
	Calcium-mediated signalling	C2 domain-containing protein
	Unknown	Coiled-coil domain-containing protein 55
mul-miRn10-3p	Auxin signaling; Transcription regulation	Auxin response factor 19
	Transcription regulation; Development	Zinc finger family protein
	Transcription regulation	Squamosa promoter binding protein-like 14
	Transcription regulation	RNA polymerase II transcription mediators
	Signal transduction; Development	Leucine-rich receptor-like protein kinase family protein
	Signaling pathway	Protein kinase superfamily protein
	Metabolic process	S-formylglutathione hydrolase
	Metabolic process;	Esterase/lipase/thioesterase family protein
	Metabolic process; Response to stress	Aconitase 3
	Response to stress	AWPM-19-like family protein
	Unknown	Uncharacterized protein

**Table 5:** Predicted targets for the differential novel miRNAs.

MiRNA-name	Putative GO process	Predicted target annotations in mulberry transcriptome data
mul-miRn21-3p	Signal transduction	Transducin/WD40 repeat-like superfamily protein
	RNA processing	Tetratricopeptide repeat (TPR)-like superfamily protein
mul-miRn22-5p	Metabolic process	2-oxoglutarate dehydrogenase
mul-miRn23-5p	Cytokinin metabolic; Development	SOB five-like 2
	Development; Auxin homeostasis; Gibberellic acid mediated signaling pathway	Lateral root primordium (LRP) protein-related
	Transcription; Development; Gibberellin biosynthetic process	Integrase-type DNA-binding superfamily protein
	Response to auxin stimulus	SAUR-like auxin-responsive protein family
	Defense response	Disease resistance protein (TIR-NBS-LRR class) family
	Unknown	Calcium-dependent lipid-binding family protein
	Metabolic process	Cellulose synthase family protein
	Unknown	TPR-like superfamily protein
	Unknown	Transposable element gene
mul-miRn24-5p	Metabolic process	ATP-citrate lyase A-3
	Membrane transport	Oligopeptide transporter 1
mul-miRn28-3p	Defence response	ADR1-like 1
mul-miRn29-5p	Ehylene mediated signaling pathway; Transcription regulation	Integrase-type DNA-binding superfamily protein
	Secondary metabolitic process; Environmental responses	Cytochrome P450, family 96, subfamily A, polypeptide 5
	Transcription regulation	Tudor/PWWP/MBT domain-containing protein
	Defence response; Flavonoid biosynthetic process	2-oxoglutarate (2OG) and Fe(II)-dependent oxygenase superfamily protein
	Unknown	Transposable element gene
	Defence response	Disease resistance protein
mul-miRn30-3p	Development; Environmental responses	PPR repeat-containing protein,
	Signaling pathway	Serine/threonine-protein kinase-like protein CCR2
	Metabolic process	Oxidoreductase family protein
	Transcription regulation	SAP domain-containing protein
mul-miRn31-3p	Metabolic process; Development	ARPN | plantacyanin
	Transcription regulation	SAC3/GANP/Nin1/mts3/eIF-3 p25-family protein
mul-miRn32-5p	Response to stress; Signaling pathway	U-box domain-containing protein kinase family protein
	Signaling pathway	Leucine-rich repeat protein kinase family protein
	Signaling pathway	Protein kinase superfamily protein

### Mul-miR482a-5p accumulated in phloem sap under phytoplasma-infection is mobile

**Figure 5:**
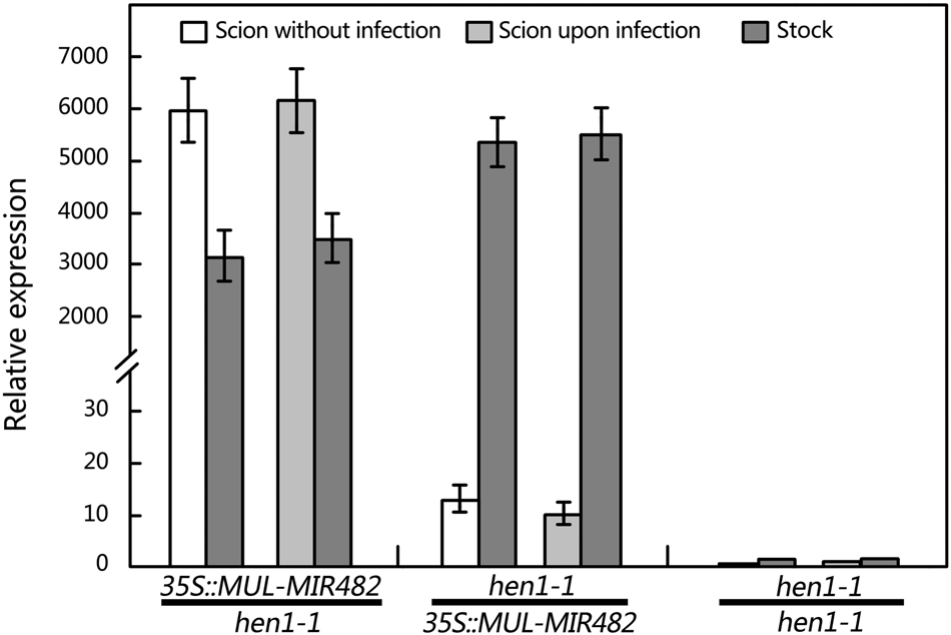
Measurement of mul-miR482a-5p in scions and rootstocks of grafted plants. Infection experiments were performed by spraying *Pst*. DC3000 suspensions at 10^8^ CFU mL^−1^ in 10 mM MgCl_2_ with 0.04% (v/v) Silwet L-77 onto leaves of scions. Mul-miR482a-5p abundance was detected by RT-qPCR. The relative miRNA abundance was evaluated using comparative Ct method taking U6 as a reference. Values are given as the mean ± SD of three experiments in each group.

It was reported that some miRNAs accumulated in the phloem sap are translocatable^51^. Our data showed that the mul-miR482a-5p accumulated strongly in the mulberry phloem sap under phytoplasma infection, so it is reasonable to suspect that it was mobile in the phloem sap. To investigate whether mul-miR482a-5p was mobile in the phloem, we performed grafting experiments using mul-miR482a-5p-overexpression and *hen1-1* mutant Arabidopsis thaliana. After the establishment of graft unions, different parts of the successful grafts were used to analyse the mul-miR482a-5p abundance by RT-qPCR. As expected, the translocation of mul-miR482a-5p from overexpressing scions to *hen1-1* rootstocks was observed in various independently grafted plants both with and without scions suffering *Pseudomonas syringae* pv. tomato DC3000 (*Pst*. DC3000) infection. However, little mul-miR482a-5p was detected in the *hen1-1* scions grafted with mul-miR482a-5p overexpressing rootstock for scions with or without *Pst*. DC3000 infection (Fig. 5). This result indicates that mul-miR482a-5p can be transported efficiently across the graft junction from scions to rootstock under both infective and uninfective conditions. However, mul-miR482a-5p can scarcely be transported in the opposite direction.

**Figure 6:**
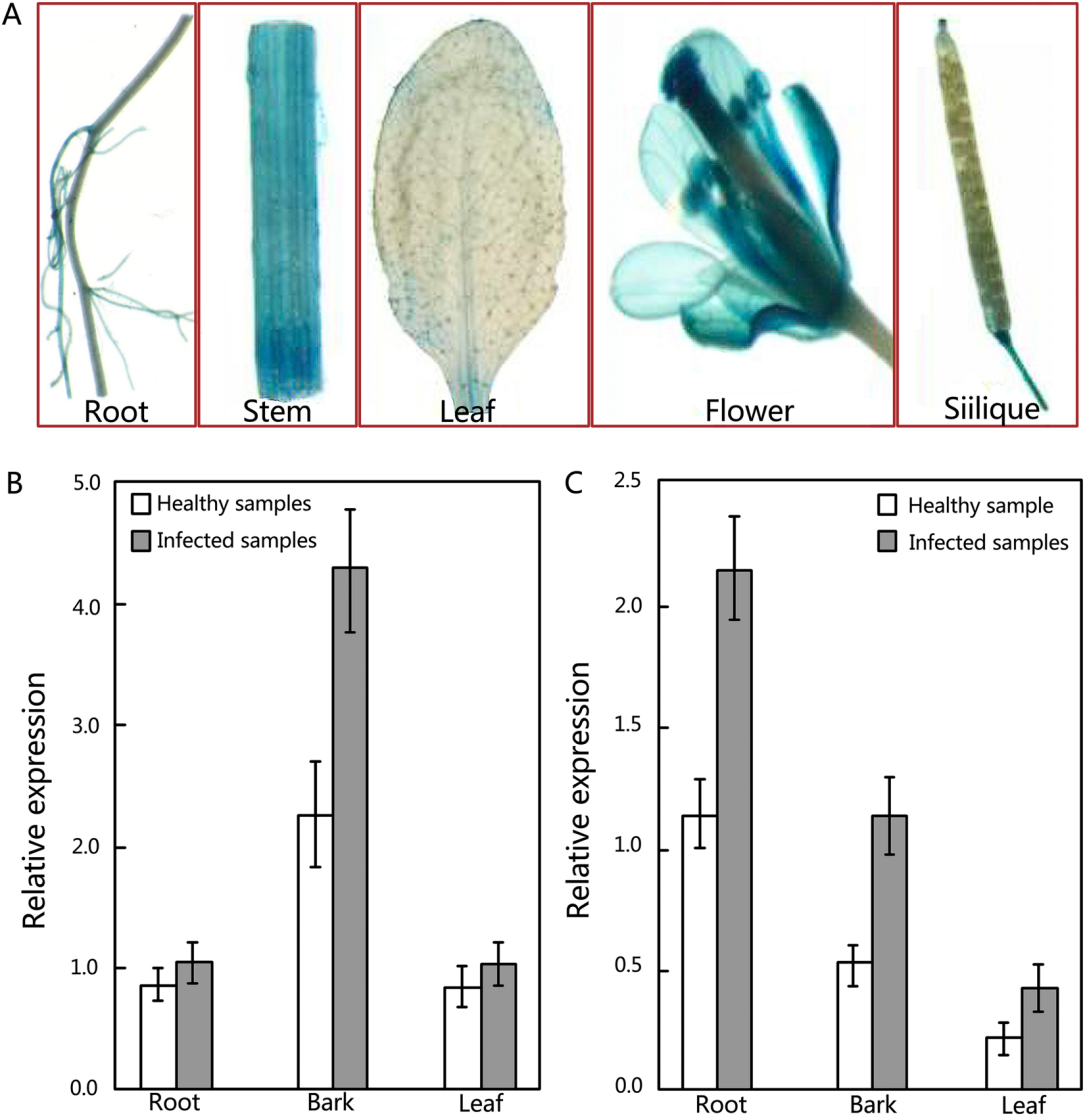
Tissue localization of *MUL-MIR482A* and measurement of pri-mul-miR482a and mul-miR482a-5p. (**A**) GUS staining in *MUL-MIR482* promoter::reporter transgenic plants. (**B** and **C**) Measurement of pri-mul-miR482a (**B**) and mature mul-miR482a-5p (**C**) in various tissues of mulberry, respectively. Pri-mul-miR482a and mul-miR482a-5p abundance were detected by RT-qPCR, and relative abundance was evaluated using comparative Ct method using actin (Accession No. DQ785808) and U6 as the reference, respectively. Values are given as the mean ± SD of three experiments in each group.

To provide further evidence that the mul-miR482a-5p is mobile, the promoter of *MUL-MIR48*2*A-5p* was cloned and fused to the reporter gene encoding β-glucuronidase (GUS) to analyse the expression pattern of mul-miR482a-5p in various tissues. Staining results showed that GUS activity was predominantly observed in stems and flowers, and the reporter signal in roots was very low, with no signal detected in the leaves and siliques (Fig. 6A). Corresponding to the staining results, when the pri-mul-miR482a transcript was examined in mulberry, more pri-mul-miR482a transcript was detected in the stem than in the root (Fig. 6B). However, this did not result in higher accumulation of mature mul-miR482a-5p in the stem than the root. In contrast, more mature mul-miR482a-5p accumulated in the root than the stem (Fig. 6C). The opposite accumulation of pri-mul-miR482a and mature mul-miR482a-5p in stems and roots implies that *MUL-MIR482A* was highly expressed in stem and mature mul-miR482a-5p was transported to roots. However, the abundance of mature mul-miR482a-5p increased in the infected roots compared to healthy roots (Fig. 6C), and the abundance of mul-miR482a-5p primary transcripts did not increase in the infected roots (Fig. 6B). Therefore, mul-miR482a-5p, but not its precursors, is mobile within the phloem. Although the level of mul-miR482a-5p increased in the infected stem barks, the abundance of mul-miR482a-5p did not increase in the infected leaves (Fig. 6C). This may confirm that mul-miR482a-5p can be transported from upper to lower parts but can scarcely be transported in the opposite direction.

### Mul-miR482a-5p accumulated in phloem sap has physiological functions

**Figure 7:**
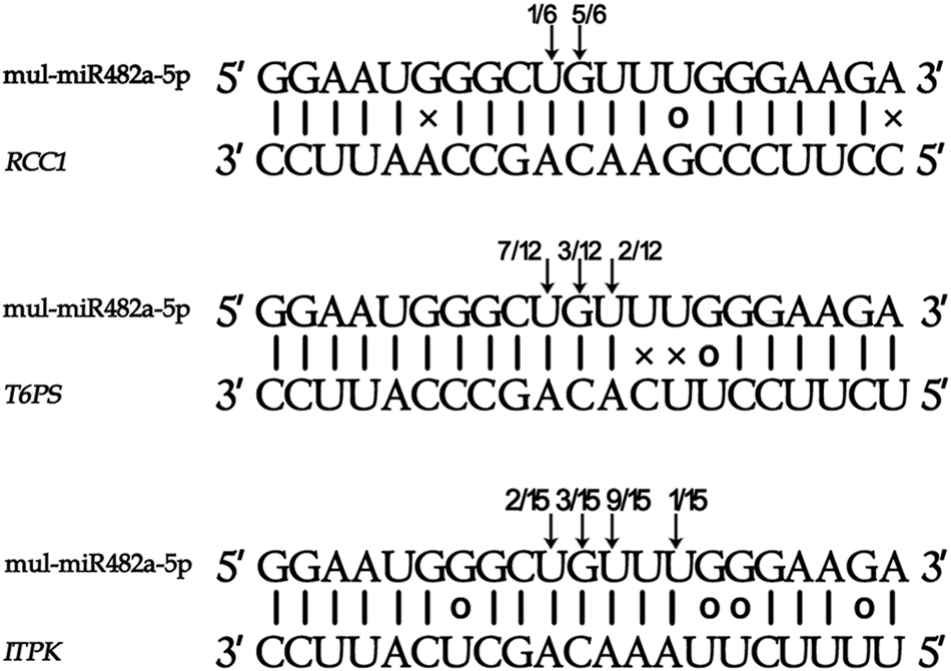
Validation of predicted target genes of mul-miR482a-5p using 5′ RLM-RACE. The mul-miR482a-5p cleavage sites on its target genes were highlighted with an arrow. The number is the frequency of accurate clones when validating cleavage sites of target mRNAs. RCC1, regulator of chromosome condensation family protein gene. T6PS, trehalose 6-phosphate synthase gene. ITPK, inositol 1,3,4-trisphosphate 5/6-kinase family protein gene.

**Figure 8:**
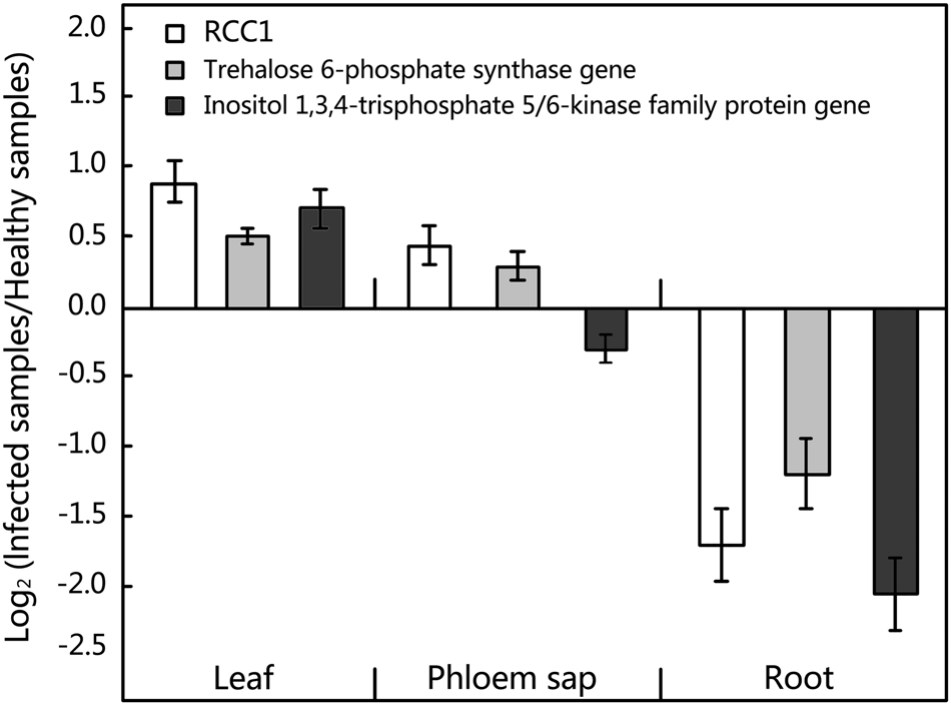
Abundance analysis of predicted target genes of mul-miR482a-5p by RT-qPCR. Relative gene expression was evaluated using comparative Ct method with actin (Accession No. DQ785808) as the reference gene. Log2 values of the ratio of phytoplasma-infected samples to healthy samples are plotted. Values are given as the mean ± SD of three experiments per group.

The regulator of chromosome condensation family protein (RCC1) gene, trehalose 6-phosphate synthase gene and inositol 1,3,4-trisphosphate 5/6-kinase family protein gene were predicted as targets of mul-miR482a-5p and were experimentally verified by 5′-RLM RACE analyses (Fig. 7). To examine whether the translocation of mul-miR482a-5p in the phloem had physiological functions, the expression levels of its target genes in leaves, phloem saps, and roots were analysed by RT-qPCR (Fig. 8). The results showed that the expression levels of the three target genes have no significant change between infected and healthy leaves. This was consistent with the level of mul-miR482a-5p, which did not differ between infected and healthy leaves. The expression levels of the three target genes showed no significant change between infected and healthy phloem sap, although the level of mul-miR482a-5p was significantly increased in the infected phloem sap. This may be because there was no RNase, which was necessary for the cleavage of target mRNAs. Therefore, mul-miR482a-5p may have no physiological function that directs cleavage of its target mRNAs in the phloem sap. However, the expression of all three target genes was down regulated in the infected roots compared to healthy roots, and this coincided with the changes in mul-miR482a-5p in the roots. Thus, the translocation of mul-miR482a-5p in the phloem might have physiological cleavage functions for its target genes in the roots.

**Figure 9:**
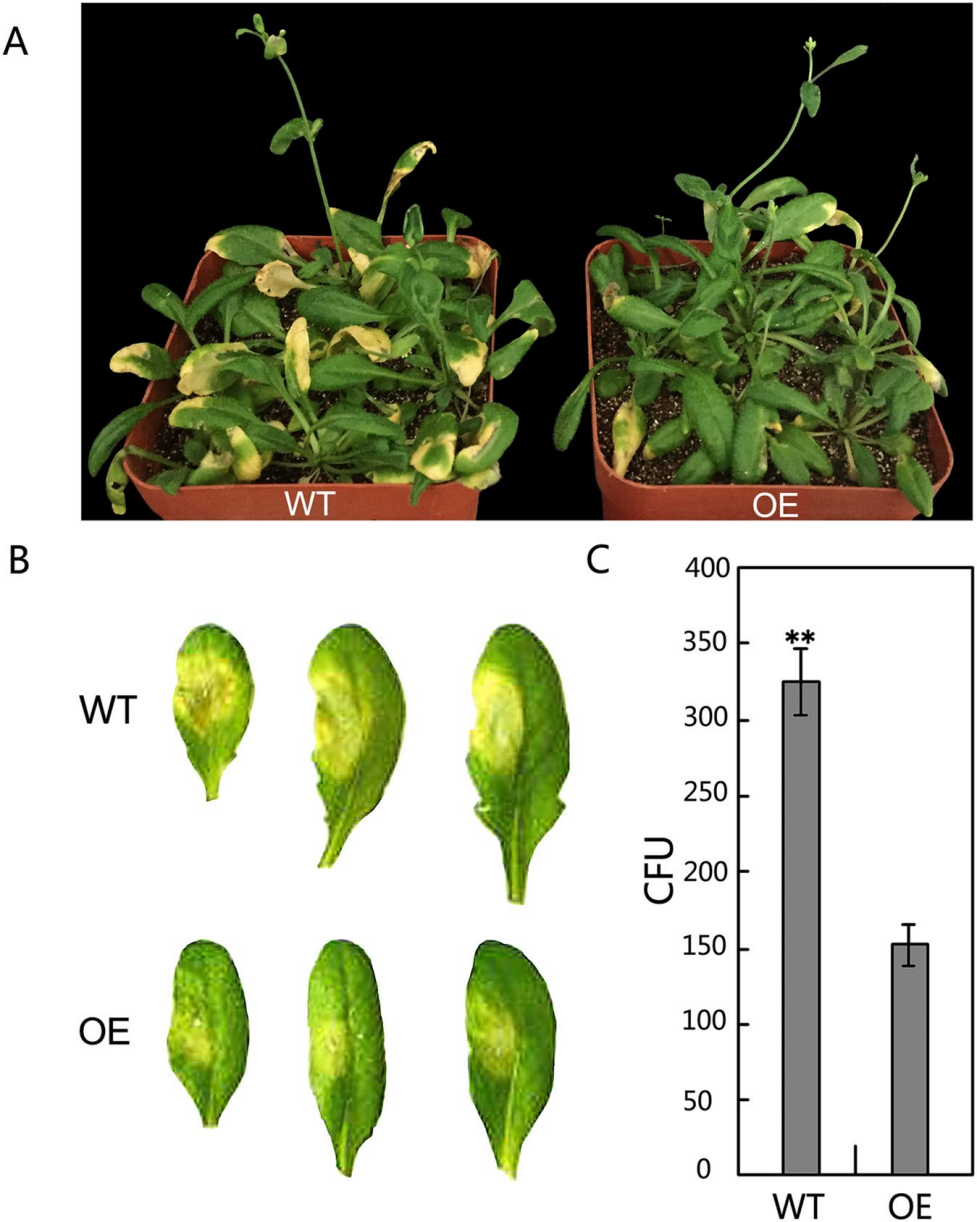
Analysis of resistance of transgenic Arabidopsis plants to *Pst*. DC3000. (**A**) Phenotypes of plants spray-inoculated with *Pst*. DC3000. (**B**) Phenotypes of leaves vacuum-infiltrated with *Pst*. DC3000; disease symptoms were recorded using a camera 3 days after inoculation; (**C**) Colony-forming units (CFU) of *Pst*. DC3000 in infected *Arabidopsis* leaves. Bacterial numbers were calculated at 3 days after inoculation and represented as CFU per gram leaf tissue, and CFU of *Pst*. DC3000 in infected leaves was counted in a 1/1000-fold bacterium solution. Bioassays were performed three times, each with three replicates, and each value is mean ± SD of three experiments. Asterisks indicate significant difference based on Student’s t-test (**P < 0.01). WT, Wild type Arabidopsis Col-0; OE, Transgenic *RCC1* Arabidopsis plants.

To further explore the physiological functions of mul-miR482a-5p, one of the target genes, *RCC1*, was also cloned and transformed into Arabidopsis thaliana, and *RCC1*-overexpressing and wild type Arabidopsis thaliana were inoculated with *Pst*. DC3000. The results showed that the *RCC1* transgenic Arabidopsis plants showed stronger resistance to *Pst*. DC3000 than wild-type plants, suggesting that the *RCC1* gene may be a positive regulator of defence responses (Fig. 9). Therefore, mul-miR482a-5p may repress the expression of *RCC1* in infected mulberry and reduce host resistance to biotic stress.

## Discussion

### Component complexity of miRNAs in phloem sap

Although a number of miRNAs have been previously identified in the phloem sap of several herbaceous plants, to the best of our knowledge, no data on phloem miRNAs is available for woody perennials except for apple. Our study demonstrates that mulberry phloem sap contains small RNAs that are major contributors to the phloem sap RNA population. Some highly expressed miRNAs such as miR166, miR167, and miR172 identified in mulberry phloem sap were also detected in the phloem sap of *B. napus*^37^, apple (*M. domestica* “Royal Gala”)^39^, and pumpkin (*Cucurbita maxima*)^40^, suggesting that some miRNAs were conserved across plant species. However, some miRNAs, such as miR171, which were detected in mulberry, *B. napus*^37^ and pumpkin^40^ phloem sap were not detected in apple phloem sap. Meanwhile, miR403 and miR162 were detected in *B. napus*^37^, apple^39^, and pumpkin^40^ phloem sap but were not present in mulberry. In addition, some novel miRNA candidates were identified in our data. Therefore, the compositions of phloem sap miRNAs differed between herbaceous and woody plants and even among woody species. Furthermore, our data showed that 43 miRNAs were differentially expressed in mulberry phloem sap in response to phytoplasma-infection. It was also reported that the composition of phloem sap miRNAs differed under different nutrient stresses. Thus, the miRNA composition of phloem sap is complex, and identification and characterization of the phloem miRNAs of mulberry may enhance current knowledge of the miRNA composition of phloem sap and help to discover new candidates that have significant action on phloem functions.

### MiRNAs in phloem sap and leaves are distinct in complement and expression pattern in response to phytoplasma infection

**Figure 10:**
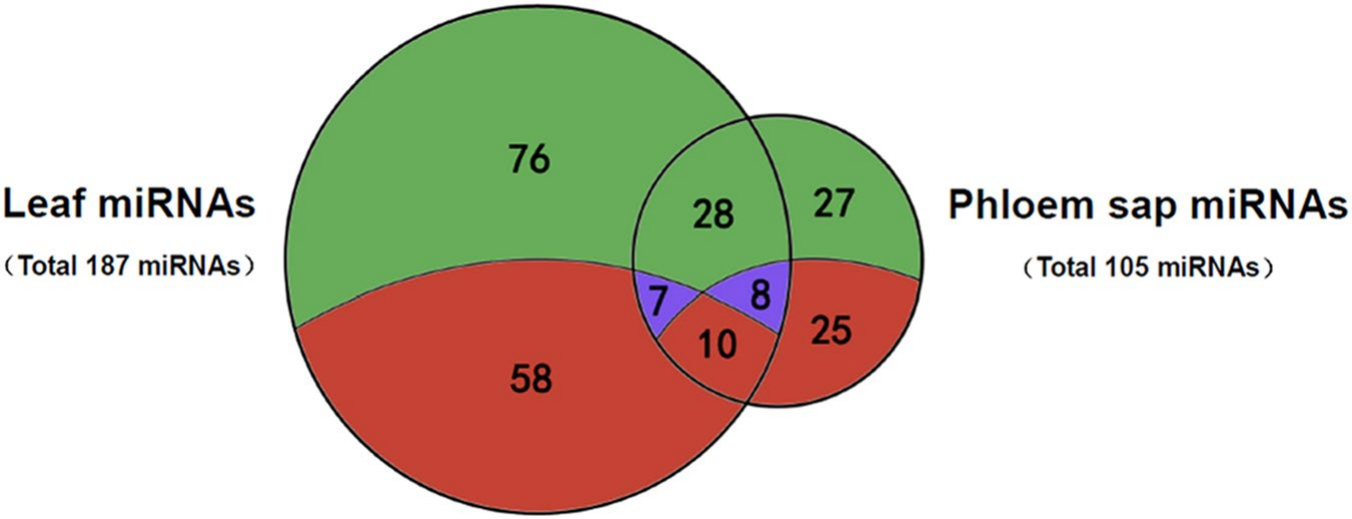
Venn diagram indicating miRNA identification profiles in phloem sap and leaves from mulberry. Values in green and red sections represent numbers of undifferentially and differentially expressed miRNAs. Values in blue sections represent numbers of miRNAs differentially expressed specifically in phloem sap or leaves.

Plant miRNAs often show differential expression among various tissues^39^, and many miRNAs present in the phloem sap of mulberry were identified in this study. When the identified miRNAs were compared to previously published collections of miRNAs in the leaves of mulberry in the response to phytoplasma infection^31^, there were 52 miRNAs identified in the phloem sap but not the leaves, and 134 miRNAs were identified in the leaves but not the phloem sap (Fig. 10). Among the 53 miRNAs common to the leaves and phloem sap, the relative levels of expression of some miRNAs, such as mul-miR2199, mul-miR2916, mul-miR5813 and mul-miR6300, were high in the phloem sap but low in the leaves. This demonstrates that phloem sap contains a specific set of miRNAs distinct from leaves and that a set of phloem-enriched sRNAs exists. Moreover, among the 43 phytoplasma-responsive miRNAs identified in phloem sap, only 10 miRNAs were expressed differently in healthy and infected leaves. This may be because not all miRNAs in phloem sap can be translocated, and mobile miRNAs might be translocated in different directions. Therefore, the expression pattern of miRNAs in phloem sap was distinct from leaves in response to phytoplasma infection, and different miRNAs might have distinct localizations and functions. Interestingly, the 10 common phytoplasma-responsive miRNAs between phloem sap and leaves showed the same expression pattern. These miRNAs could potentially act as a long-distance information transmitters in response to phytoplasma infection in mulberry. Further experiments are required to uncover the translocatability and functions of these miRNAs in modulating the response of mulberry to phytoplasmas.

### Role of miRNAs in phloem sap

Although many miRNAs have been detected in many plant phloem saps, only a few miRNAs have been shown to translocate between cells and over long distances^42–44,47^. It is not clear whether all differentially expressed miRNAs in the phloem sap are mobile. Since phytoplasmas are restricted to sieve elements of phloem tissues, the sieve elements may experience phytoplasma infection earlier than other tissues. Thus, the translocated phytoplasma-responsive miRNAs in phloem sap might have roles in long-distance signalling in response to phytoplasma infection and serve to coordinate physiological responses with other plant parts that are not yet infected. Even if some differentially expressed miRNAs in the phloem sap were not mobile, it was reported that the miRNAs in the phloem sap may act to prevent translation and movement of their target mRNAs^39^. Although the putative target genes were bioinformatically predicted, the role of differentially expressed miRNAs to prevent translation and movement of their target mRNAs, which may be involved in signalling in response to phytoplasma infection, remains to be studied. The elucidation of the roles of these phytoplasma-responsive miRNAs in the phloem sap may reveal the mechanisms underlying the interactions of phytoplasma and mulberry.

The most typical symptoms of phytoplasma diseases indicate perturbations in plant hormonal balance^52–55^. In this study, we also found expression changes for several phloem sap miRNAs involved in auxin signalling and auxin metabolism, e.g., differentially expressed mul-miR319a was predicted to target the MYB transcription factor, which redirects auxin signal transduction by interacting with ARFs and plays a role in plant hormone responses^27^. Differential mul-miR1223e was predicted to target genes coding for O-fucosyltransferase family protein and SAUR-like auxin-responsive protein, which are associated with auxin metabolism (Table 4). In addition to the auxin signalling pathway, mul-miR1223e was found to target the genes involved in salicylic acid signalling, and mul-miR157a was predicted to target the gene coding galactose oxidase/kelch repeat superfamily protein associated with brassinosteroid biosynthesis. Meanwhile, mul-miR529b was found to target the gene involved in abscisic acid and jasmonic acid signalling pathways. In addition, mul-miR894 and mul-miRn25-5p were found to be associated with the ethylene signalling pathway, and mul-miRn23-5p was predicted to target the lateral root primordium (LRP) protein-related gene and SOB five-like 1 gene, which was related to the gibberellic acid-mediated signalling pathway and cytokinin metabolic processes. These data are consistent with earlier reports that show phytoplasma infection-induced alteration in hormonal signalling leading to symptoms in infected plants.

Symptoms induced in infected plants suggest that phytoplasma infection may modulate developmental processes within the plant host^56^. Our data showed that some phytoplasma-responsive miRNAs target the transcription factors involved in development. For example, mul-miR156a was predicted to target the Squamosa Promoter-Binding Protein-Like (SPL) family, which plays important roles in flower and fruit development, plant architecture and phase transition in plants^57,58^. Meanwhile, mul-miR319a was predicted to target the MYB transcription factor, which was crucial to the control of proliferation and differentiation in a number of cell types and key factors in regulatory networks controlling development^59^. In addition, our results showed differentially expressed miRNAs—such as mul-miR1223e, mul-miR165b-5p, mul-miR529, mul-miRn23-5p—that target the genes associated with modulating plant development through various pathways. The changes in these miRNAs in the phloem sap may disorder the expression of many genes involved in diverse development processes, causing symptoms of phytoplasma disease in the infected plants.

As intracellular parasites, phytoplasmas have lost many metabolic genes and must obtain essential metabolites from their hosts^60^, which has a great impact on the metabolome of infected plants^7,14,52^. Our results showed that phytoplasma infection alters the profiles of a number of miRNAs involved in metabolism in phloem sap. These differentially expressed miRNAs target genes associated with protein metabolism, CHO metabolism and lipid metabolism. Furthermore, some miRNAs targeting the genes associated with secondary metabolism were also differentially expressed. For example, mul-miR156a and mul-miR529-3p were predicted to target UDP-glucosyltransferase genes, which were involved in the flavonoid biosynthesis pathway^61^. Therefore, differentially expressed miRNAs in the phloem sap may disturb many metabolic processes and have a significant effect on the response against phytoplasmas.

### Some phytoplasma-responsive miRNAs modulate overlapping signalling of biotic and abiotic stresses

Plants have evolved sophisticated mechanisms to sense and respond to diverse biotic stresses^62^. Our miRNA expression analysis showed many differentially expressed miRNAs targeting genes associated with defence response genes. The changes in these miRNAs may down-regulate or up-regulate their target gene expression and alter plant resistance to phytoplasma. Interestingly, we also found that some differentially expressed miRNAs were responsive to abiotic stresses. For example, mul-miR397a were predicted to target the gene casein kinase II beta chain 3, which has roles in response to light stimulus response^63^, and mul-miR529b, which was predicted to target the 3-ketoacyl-CoA synthase 19 gene involved in response to cold stress^64^. In addition, mul-miR529-3p was predicted to target the ARM repeat superfamily protein associated with salt stress and shoot gravitropism^65^. Meanwhile, several phytoplasma-responsive miRNAs were reported to be differentially expressed under various abiotic stresses in other plant species. For instance, miR156, miR157, and miR390 were reported to be responsive to salt, drought, cold, and heat stress in many other plants^29,62^ and were detected to be differentially expressed in phloem sap in response to phytoplasma infection in this study. This may be because phytoplasma infection had a great influence on the growth and development of mulberry, and these miRNAs may contribute to modulation of the necessary growth and developmental adjustments to adapt to phytoplasma-infected conditions. In conclusion, our results suggested that phytoplasma infection may cause both biotic and abiotic stress in the mulberry, and some phytoplasma-responsive miRNAs involved in both biotic and abiotic stress signalling converge upstream of phytoplasma infection. The infected plant can modulate protective responses by controlling the abundance of these miRNAs via overlapping signalling. However, further experiments are required to uncover signalling in the phloem sap that modulates the response of mulberry to phytoplasmas.

### Mul-miR482a-5p might negatively regulate mulberry resistance to phytoplasma-infection

The miR482 superfamily is a group of plant-specific miRNAs targeting the NBS-LRR gene, and several reports have demonstrated that the miR482-NBS-LRR regulatory loop is part of the immune response induced by pathogens^66–69^. Moreover, miR482 was also found to participate in guidance for the biosynthesis of secondary phasiRNAs, which are involved in controlling immune-response genes^66,70^. To date, miR482 has been confirmed to be distributed in more than 20 species, and approximately sixty primary transcripts of miR482 have been identified. Among the primary transcripts identified, there are approximately 20 primary transcripts that can be processed to generate both miR482-5p and miR482-3p (http://www.mirbase.org). To date, there are many reports of miR482-3p, but the function of miR482-5p is still not well understood. Plants infected with pathogens showed a reduced level of miR482 and an increased level of miR482 target mRNAs, suggesting that the miR482-mediated silencing cascade is suppressed by pathogen attack and may be a defence response of plants^71^. However, the level of mul-miR482a-5p, not mul-miR482a-3p, was changed significantly in the phloem sap infected by phytoplasma (Table 1). Mul-miR482a-5p was predicted to target the *RCC1* gene, which is the guanine nucleotide exchange factor for the nuclear GTP binding protein Ran, and is probably involved in various biological processes, but the role of this gene under stress is currently not clear^72^. Our results showed that the *RCC1* gene may be a positive regulator of defence responses (Fig. 9). Therefore, the up-regulation of mul-miR482a-5p may repress the expression of *RCC1* in the infected plant and reduce host resistance to phytoplasma. This is consistent with the report that RCC1 family proteins were down-regulated in the incompatible interaction between soybean and *Phytophthora sojae*^73^. It was suggested that nucleocytoplasmic trafficking plays an essential role in the expression of disease resistance^74^, and nuclear localization of some disease resistance (R) proteins, such as members of CC-NB-LRR and TIR-NB-LRR proteins, is essential for their resistance function^75,76^. As RCC1 plays a major role in nucleocytoplasmic transport^72^, silencing of *RCC1* may result in partial impairment of nucleocytoplasmic trafficking and loss of resistance function of some disease resistance (R) proteins. Thus, when mulberry plants were infected by phytoplasma, the mul-miR482a-3p did not decrease, suggesting that the miR482-NBS-LRR regulatory loop was not induced. Moreover, increased mul-miR482a-5p might repress the resistance to phytoplasma mediated by RCC1. Therefore, phytoplasma-derived suppression of RNA silencing may repress the whole host immune system during infection and potentially enhance phytoplasma colonization and amplification. However, further research is required to elucidate the regulatory mechanisms of the *RCC1* gene.

In conclusion, the characterization of miRNA-Seq-based expression profiling of miRNAs allowed for the identification of many phytoplasma-responsive miRNAs in mulberry phloem sap. Future investigation will explore the functions and regulatory networks of these miRNAs. The information provided here will be particularly useful for a complete understanding of the function of miRNAs in phloem sap and will lay the foundation to reveal the mechanisms underlying phytoplasma pathogenicity.

## Methods

### Plant material

One-year-old cutting seedlings collected from Husang 32 (*Morus multicaulis* Perr.) were used as rootstock and grafted to scions from healthy or phytoplasma-infected mulberry trees (Husang 32). All establishment graft unions were incubated in a greenhouse, and plants showing Witches’ broom disease symptoms were confirmed by PCR assay with an amplified fragment of the phytoplasma 16S rRNA gene (GenBank Accession No. EF532410) using the primers (P16mF: 5′-TAAAAG ACCTAGCAATAGG-3′ and P16mR: 5′-CAATCCGAACTGAGACTGT-3′) as previously described^53^.

### Phloem saps collection and purity assessing

Phloem sap was collected from infected and healthy mulberry plants using the shoot exudation method^77^. First, shoots were excised with a sterile razor blade, the first droplets were discarded, and the cut surface was blotted with sterile filter paper several times to avoid contamination. Exuding phloem sap was collected into an Eppendorf tube containing TRIzol reagent (Invitrogen, Carlsbad, CA, USA). Leaf and phloem sap RNAs were isolated using a TRIzol kit (Invitrogen) following the manufacturer’s instructions. To detect *RuBisCO* and *MmPP16* transcripts, cDNA was synthesized using oligo (dT)18 primer (GACTCTAGACGACATCGA(T)15) and ReverTra Ace M-MLV RTase (TaKaRa, Dalian, China). RT-PCR assays were performed in 25-µl reaction volumes containing 20 ng cDNA and 150 nM forward and reverse primers. The primers used for amplification of *RuBisCO* and *MmPP16* genes are shown in Supplementary Table 1.

### Small RNA library construction and high-throughput sequencing

Isolated phloem sap RNAs were used to prepare a small RNA library according to the protocol of the TruSeq Small RNA Sample Prep Kits (Illumina, San Diego, USA). Single-end sequencing (36 bp) was performed on an Illumina Hiseq2500 instrument following standard protocols. Three independent libraries each (biological replicates) were analysed for infected and healthy phloem saps.

### MiRNAs identification

The raw sequences tags obtained from HiSeq sequencing were cleaned to remove adapter dimers, junk, low complexity, common RNA families (rRNA, tRNA, snRNA, snoRNA) and repeats, and the length distribution of the clean tags was summarized. The trimmed reads longer than 18 nt were annotated into different categories, and the sequences of 18–25 nucleotides were compared to a miRBase database v21.0 (http://www.mirbase.org/). The sequences with identical or related sequences from other plants were regarded as conserved miRNAs. All remaining unmapped sequences were BLASTed against our mulberry transcriptome database, and the hairpin RNA structures containing sequences were predicted using RNAfold software and used to predict novel miRNAs using Mireap (http://sourceforge.net/projects/mireap/).

### Differential expression analyses of miRNAs

The frequency of miRNA was normalized by the total number of miRNAs in every sample, where normalized expression = (Actual miRNAs sequencing reads count/Total clean reads count) × 1,000,000. The fold change between infected (IPS) and healthy phloem sap (HPS) was calculated as follows: fold-change = log2(IPS/HPS). Statistical analysis was performed according to Poisson distribution, and the P value was calculated based on the formula:

$$\begin{array}{llll}{\rm{P}}(x|y) & = & {(\frac{{{\rm{N}}}_{2}}{{{\rm{N}}}_{{\rm{1}}}})}^{{\rm{y}}}\frac{(x+y)!}{{\rm{x}}!{\rm{y}}!{(1+\frac{{{\rm{N}}}_{2}}{{{\rm{N}}}_{1}})}^{{\rm{x}}+{\rm{y}}+1}} & C(y\le {{\rm{y}}}_{\min }|X)=\sum _{{\rm{y}}=0}^{{\rm{y}}\le {{\rm{y}}}_{\min }}p({\rm{y}}|{\rm{x}})\\  &  &  & D(y\ge {{\rm{y}}}_{\max }|X)=\sum _{{\rm{y}}\ge {{\rm{y}}}_{\max }}^{\infty }p({\rm{y}}|{\rm{x}})\end{array}$$

N_1_ and N_2_ represent the total count of clean reads of a given miRNA in the sRNA library of infected and healthy phloem sap, respectively. The x and y represent normalized expression levels of a given miRNA in the sRNA library of infected and healthy phloem saps, respectively.

A fold-change ≥ 2 and P ≤ 0.05 were used as criteria to identify differentially expressed miRNAs, and an miRNA was designated as significantly differentially expressed if its expression value varied more than two-fold and P ≤ 0.05 between infected and healthy phloem saps.

### Target prediction of differential miRNAs

The target genes of the differentially expressed miRNAs were predicted using the software psRNATarget (http://plantgrn.noble.org/psRNATarget/) by submitting miRNA sequences to a search against our in-house mulberry transcriptome data following the criteria of (i) maximum expectation less than 3.0; (ii) multiplicity of target sites 2; (iii) range of central mismatch for translational inhibition 9–11 nt; and iv) maximum mismatches at the complementary site ≦ 4 without any gaps. All predicted target genes were aligned with the reference *Arabidopsis thaliana* database downloaded from TAIR (http://www.arabidopsis.org/; version TAIR10) to annotate their functions, and the GO terms of these targets were also annotated based on their TAIR GO categories.

### Quantitative real-time PCR analysis for miRNAs and mRNAs

RNA was extracted using the TRIzol^®^ reagent following the manufacturer’s recommendations (Invitrogen, Carlsbad, CA, USA) and digested with DNase I. Real-time PCR analyses for miRNAs and mRNAs were performed using the PrimeScriptTM miRNA qPCR Starter Kit Ver.2.0 (TaKaRa, Dalian, China) and the SYBR Premix Ex TaqTM kit (TaKaRa, Dalian, China) on the Rotor-Gene 3000A system (Bio-Rad, Munich, Germany), respectively, according to the manufacturer’s protocol for the Rotor-Gene 3000A system. The U6 and actin genes were used as reference genes for miRNA and mRNA normalization, respectively. The U6 gene was amplified using the primer (5′-ATGGCCCCTGCGTAAGGATG-3′), and actin was amplified using primer pair (F: 5′-CAGTGCTTCTCACTGAGGCTC-3′ and R: 5′-GGAAGAGGACTTCTGGGCATC-3′). The primers (Supplementary Tables 1, 2) used to amplify the genes and miRNAs were designed based on our available mulberry transcriptome data. The relative expression levels of miRNA and mRNA were evaluated using the Comparative cycle threshold (Ct) method^78^. All samples were assayed in triplicate.

### Target validation

For miRNA target validation, a modified gene-specific 5′ RNA ligase-mediated rapid amplification of cDNA ends (5′ RLM-RACE) was performed using the GeneRacer Kit (Invitrogen, Carlsbad, CA, USA). Total RNA was isolated from mulberry seedlings using the TRIzol kit (Invitrogen, Carlsbad, CA, USA) following the manufacturer’s instructions, ligated to the RNA oligo adapter (5′-CGACUGGAGCACGAGGACACUGACAUGGACUGAAGGAGUAGAAA-3′) and reverse transcribed with SuperScript III reverse transcriptase using oligo(dT) primer. The resulting cDNA was PCR-amplified with GeneRacer 5′ primer (5′-CGACTGGAGCACGAGGACACTGA-3′) and each respective gene-specific outer primer (shown in Supplementary Table 3). The PCR product was further amplified by nested PCR using GeneRacer 5′ nested primer (5′-GGACACTGACATGGACTGAAGGAGTA-3′) and each respective gene-specific inner primer (shown in Supplementary Table 3). The final PCR product was gel-purified and cloned into a pMD18-T vector (Invitrogen, Carlsbad, CA, USA) for sequencing.

### Micrografting experiments

Four-day-old seedlings of *hen1-1* mutant and transgenic Arabidopsis thaliana were used for micrografting experiments. The seedlings were cut transversely using a sterile razor blade and combined inside silicon tubing (0.3 mm internal diameter). The graft unions were cultured on 1.5% (w/v) agar plates with half-strength MS medium for 9 days and were hydroponically cultured for 10 d. Grafted plants without adventitious roots were selected, and the scions and stocks of the selected grafted plants were harvested for RNA isolation. Then, the RNAs were used for RT-qPCR of the miRNA. To investigate whether the miRNA moves during infective conditions, the leaves of the scions of graft unions were spray-inoculated with *Pst*. DC3000 suspensions at 10^8^ CFU mL^−1^ in 10 mM MgCl_2_ with 0.04% (v/v) Silwet L-77. Three days after inoculation, the RNAs of the scions and stocks of the grafted plants were isolated and used for RT-qPCR of the miRNA.

### Promoter activity analysis

The promoter was cloned using a Tail-PCR method and ligated into the vector pBI121 to replace the cauliflower mosaic virus (CaMV) 35S promoter and fused to the GUS (β-glucuronidase) reporter gene to create the promoter expression vector pMIR482::GUS. The derived construct vector was introduced into *Agrobacterium tumefaciens* strain GV3101, and the WT Arabidopsis plants were transformed with a floral dipping method. Histochemical staining for GUS activity was performed referring to the previously described method^79^.

### Detection of resistance against *Pst*. DC3000

The *RCC1* gene coding sequence was cloned and ligated into the vector pBI121, and the derived construct vector was introduced into *A. tumefaciens* strain GV3101 under the control of 35S. The WT Arabidopsis plants were transformed with floral dipping method. After transformation, the transformed plants were selected. Four-week-old transgenic and wild-type Arabidopsis seedlings were spray-inoculated with *Pst*. DC3000 suspensions at 10^8^ CFU mL^−1^ in 10 mM MgCl_2_ with 0.04% (v/v) Silwet L-77 or vacuum-infiltrated with bacterial suspensions at 10^7^ CFU mL^−1^ with a syringe. Three days after inoculation, disease symptoms were recorded using a camera, and bacterial numbers were calculated and represented as colony-forming units (CFU) per gram leaf tissue in a 1/1000-fold bacterium solution. Bioassays were performed three times, with three replicates each.

### Data Availability

The datasets analyzed during the current study are available from the corresponding author on reasonable request.

## Electronic supplementary material

Supplementary table 1

Supplementary table 2

Supplementary table 3

Supplementary figure 1
