# Supplementary material for: MiRNA-seq-based profiles of miRNAs in mulberry phloem sap provide insight into the pathogenic mechanisms of mulberry yellow dwarf disease
Source: Sci Rep. 2018 Jan 16;8:812. doi: 10.1038/s41598-018-19210-7 (PMC5770470; doi:10.1038/s41598-018-19210-7)
Supplement: Supplementary file 1 — Supplementary table 1 [file 41598_2018_19210_MOESM1_ESM.pdf]

MiRNA-seq-based profiles of miRNAs in mulberry phloem sap provide insight into the pathogenic mechanisms of mulberry yellow dwarf disease

Ying-Ping Gai[1*], Huai-Ning Zhao[2*], Ya-Nan Zhao[1], Bing-Sen Zhu[1], Shuo-Shuo Yuan[2],

Shuo Li[2], Fang-Yue Guo[2], Xian-Ling Ji[1,2]

Supplementary table 1. Primers used RT-PCR and RT-qPCR for mRNA abundance analysis.

Gene	Forward primer (5′-3′)	Reverse primer (5′-3′)
Actin	**CAGTGCTTCTCACTGAGGCTC**	**GGAAGAGGACTTCTGGGCATC**
MmPP16	**GGACCCGATTTCCTGTTCTG**	**ATGTCAGAGATTACCTCAGGC**
RuBisCo	**AATGATGGTGTGACTGTGGCG**	**CAGTGAGAATAGCAATATCGTC**
Regulator of chromosome condensation family protein gene	**CTTAGCGGGATTTTGGATCTG**	**AAGATCAACGGCACTGAGCCT**
Trehalose 6-phosphate synthase gene	**GGACAGCAAATCGTCGAAGTT**	**AGCAATCCGCAGTATGCTTTC**
Inositol 1,3,4-trisphosphate 5/6-kinase family protein gene	**ACCTCAAGTTGCTTGTGGTGT**	**TGGTGTAGACTTCTTAACCGC**
Pri-mul-miR482a	**GGAAAGGGAGATTTGAGCTAC**	**AGAGAATACGGAAGGGAAAGG**
