# Supplementary material for: MiRNA-seq-based profiles of miRNAs in mulberry phloem sap provide insight into the pathogenic mechanisms of mulberry yellow dwarf disease
Source: Sci Rep. 2018 Jan 16;8:812. doi: 10.1038/s41598-018-19210-7 (PMC5770470; doi:10.1038/s41598-018-19210-7)
Supplement: Supplementary file 2 — Supplementary table 2 [file 41598_2018_19210_MOESM2_ESM.pdf]

MiRNA-seq-based profiles of miRNAs in mulberry phloem sap provide insight into the pathogenic mechanisms of mulberry yellow dwarf disease

Ying-Ping Gai[1*], Huai-Ning Zhao[2*], Ya-Nan Zhao[1], Bing-Sen Zhu[1], Shuo-Shuo Yuan[2],

Shuo Li[2], Fang-Yue Guo[2], Xian-Ling Ji[1,2]

Supplementary table 2. Forward primers used in RT-qPCR for miRNA abundance analysis. Uni-miR qPCR Primer supplied by the SYBR® PrimeScript® miRNA qPCR Starter Kit was used as reverse primer.

MiRNA-name	Primer sequence (5′-3′)
Mul-miR1223e	TTGAGATGTCATGCACCACTCTG
Mul-miR1511	ACTATGCTCTGATACCATGTTAAA
Mul-miR156a-5p	GCGTGACAGAAGAGAGTGAGCAC
Mul-miR157a	TTGACAGAAGATAGAGAGCAC
Mul-miR160a-5p	GCCTGGCTCCCTGTATGCCA
Mul-miR165b-5p	GAAGTGTTCGGATCGAGGC
Mul-miR169p-3p	GGCATATGATCATCTTGGGGCTAG
Mul-miR172a	AGAATCTTGATGATGCTGCAT
Mul-miR3630-3p	GGGAATCTCTCTGATGCA
Mul-miR391-5p	TGTCGCAGGAGAGATGGCGAA
Mul-miR397a	TCATTGAGTGCAGCGTTGATG
Mul-miR482a-5p	GGAATGGGCTGTTTGGGAAGA
Mul-miR6214	CACGACACGAGCTGACGACA
Mul-miRn21-3p	GAGCAGUGCGGAGUAGCUGAG
Mul-miRn22-5p	CAGCGAACUAAACGGGCCCU
Mul-miRn28-3p	GGACUUUAUGGACCCGUCGGUG
Mul-miRn30-3p	UCCAGAAGCAAUCGUACGGGA
Mul-miRn32-5p	GGAAUGUUGUCUGGCUCGAGG
Mul-miRn33-3p	GGGAGAAAGAGGAAAAUAGGC
Mul-miRn35-5p	GCAGAAGAGUCAGAGCUUUGA
Mul-miRn36-3p	GCUGAAGCUGGGGUGGGGCC
Mul-miRn38-3p	GACUGAAAGCGGACCUGGUGGUG
U6	ATGGCCCCTGCGTAAGGATG
