# Supplementary material for: MiRNA-seq-based profiles of miRNAs in mulberry phloem sap provide insight into the pathogenic mechanisms of mulberry yellow dwarf disease
Source: Sci Rep. 2018 Jan 16;8:812. doi: 10.1038/s41598-018-19210-7 (PMC5770470; doi:10.1038/s41598-018-19210-7)
Supplement: Supplementary file 3 — Supplementary table 3 [file 41598_2018_19210_MOESM3_ESM.pdf]

MiRNA-seq-based profiles of miRNAs in mulberry phloem sap provide insight into the pathogenic mechanisms of mulberry yellow dwarf disease

Ying-Ping Gai[1*], Huai-Ning Zhao[2*], Ya-Nan Zhao[1], Bing-Sen Zhu[1], Shuo-Shuo Yuan[2],

Shuo Li[2], Fang-Yue Guo[2], Xian-Ling Ji[1,2]

Supplementary table 3. Primers used in RLM-5′RACE.

Gene	Outer primer (5′-3′)	Inner primer (5′-3′)
Regulator of chromosome condensation family protein gene	GTGTGATACTTTCCAGTTGT	CGACTATGTCAGAAGATTGC
Trehalose 6-phosphate synthase gene	GTCTATGAACACTAACTAAGAG	ACCCCATTTACTGACTGTGT
Inositol 1,3,4-trisphosphate 5/6-kinase family protein gene	AGAGGTGCAGACAAAGATTTC	GCCTCAGTGACCAAACCATGA
