# Supplementary material for: MiRNA-seq-based profiles of miRNAs in mulberry phloem sap provide insight into the pathogenic mechanisms of mulberry yellow dwarf disease
Source: Sci Rep. 2018 Jan 16;8:812. doi: 10.1038/s41598-018-19210-7 (PMC5770470; doi:10.1038/s41598-018-19210-7)

# MiRNA-seq-based profiles of miRNAs in mulberry phloem sap provide insight into the pathogenic mechanisms of mulberry yellow dwarf disease

Ying-Ping Gai[1*], Huai-Ning Zhao[2*], Ya-Nan Zhao[1], Bing-Sen Zhu[1], Shuo-Shuo Yuan[2], Shuo Li[2], Fang-Yue Guo[2], Xian-Ling Ji[1,2]

**Supplementary figure 1. The gels used in the figure 1.**

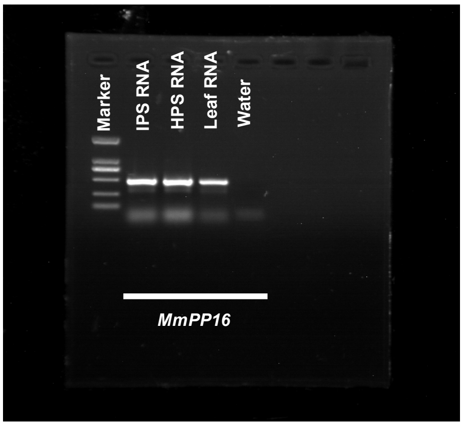


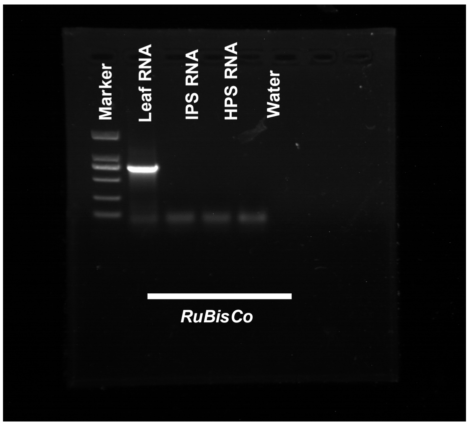

Supplement: Supplementary file 4 — Supplementary figure 1 [file 41598_2018_19210_MOESM4_ESM.pdf]
